# Silencing acetyl-CoA carboxylase A and sterol regulatory element-binding protein 1 genes through RNAi reduce serum and egg cholesterol in chicken

**DOI:** 10.1038/s41598-022-05204-z

**Published:** 2022-01-24

**Authors:** Athe Rajendra Prasad, T. K. Bhattacharya, R. N. Chatterjee, D. Divya, S. K. Bhanja, M. Shanmugam, N. G. Sagar

**Affiliations:** ICAR-Directorate of Poultry Research, Rajendranagar, Hyderabad, India

**Keywords:** Biological techniques, Biotechnology, Molecular biology

## Abstract

Cholesterol is synthesized in chicken through de novo lipid biosynthetic pathway where two most important genes viz. SREBP1 and ACACA play immense role. To minimize cholesterol synthesis, RNAi approach was adopted and accordingly, we developed transgenic chicken possessing ACACA and SREBP1 shRNA constructs, which showed lower level of ACACA and SREBP1 in serum. The serum total cholesterol, triglycerides, HDL and LDL cholesterol was significantly lower by 23.8, 35.6, 26.6 and 20.9%, respectively in SREBP1 transgenic birds compared to the control. The egg total cholesterol and LDL cholesterol content was numerically lower in both ACACA and SREBP1 transgenic birds by 14.3 and 13.2%, and 10.4 and 13.7%, respectively compared to the control. It is concluded that the protocol was perfected to develop transgenic chicken through RNAi for knocking down the expression of ACACA and SREBP1 proteins, which minimized the cholesterol and triglycerides contents in serum and eggs.

## Introduction

Chicken egg and meat are the two major sources of animal proteins for Non-vegetarian people of all strata of the society across the globe. In chicken, egg, particularly egg yolk is the major source of lipids including cholesterol and triglycerides. The LDL cholesterol is mostly deposited on the blood capillaries sometimes, causing cardiovascular disorders in human. In chicken, only 85% of de novo lipogenesis occur in liver, and the rest happens in adipose tissues. The products of de novo lipogenesis in liver are secreted in the form of VLDL and delivered to other tissues. The major factor which regulates expression of genes involved in de novo lipid biosynthesis in liver is the sterol regulatory element-binding protein (SREBP) family of transcription factors. They control cholesterol and lipid metabolism and play critical roles during adipocyte differentiation and insulin-dependent gene expression^1–4^. The SREBP family members, SREBP1 and SREBP2 are synthesized as membrane proteins in the endoplasmic reticulum (ER). The SREBPs are synthesized as large precursor proteins that are inserted into the endoplasmic reticulum membrane through two membrane-spanning domains. In the ER, the C terminus of the SREBP interacts with a protein called Scap (SREBP-cleavage activating protein), which functions as a sterol sensor activating lipid biosynthetic genes^5,6^. Thus, SREBP1 acts as transcription factor for de novo lipid biosynthetic genes regulating lipid synthesis in the body. In sterol-depleted cells, Scap transports the SREBPs from the ER to the Golgi, where they are processed by two membrane-associated proteases, the site 1 (S1P) and site 2 (S2P) proteases, which release the mature forms of the proteins^7^. These transcriptionally active fragments of the SREBPs are translocated to the nucleus, where they bind to the promoters of SREBP target genes, including genes involved in the synthesis and metabolism of cholesterol.

Another important gene involved in lipid biosynthesis is acetyl-CoA carboxylase A (ACACA). The ACACA (EC 6.4.1.2) is a complex multifunctional and biotin-containing enzyme that catalyzes the carboxylation of acetyl-CoA to malonyl-CoA, which is an intermediate substrate that plays a pivotal role in the regulation of fatty acid synthesis. Malonyl­CoA is the C_2_ donor in chain elongation process for synthesis of the very long chain fatty acids^8,9^. Thus, malonyl-CoA, generated in the lipogenic tissues by ACACA, is used as a two-carbon building block by fatty acid synthase to synthesize long chain fatty acids (LCFA). The ACACA (Molecular weight: 265 kDa) is mainly localized in lipogenic tissues such as liver and adipose tissues where fatty acids are synthesized^10^. The LCFAs are free fatty acids or non-esterified fatty acids, which are straight chain fatty acids containing twelve or more carbon atoms^11^. LCFA having carbon chain lengths of 16 and 18 constitute the major chunk of fatty acids in animal tissues where saturated 16-carbon LCFA is palmitic acid, and the saturated 18-carbon LCFA is stearic acid^11^. These LCFA suppress receptor-dependent LDL-cholesterol transport into the liver and increase production of LDL-cholesterol, which ultimately raise the plasma LDL-cholesterol concentration^11^.

In this study, we have focussed on controlling the expression of ACACA and SREBP1 genes by using RNA interference (RNAi), which is a post transcriptional gene silencing technique to degrade target mRNA in the cells. The RNAi method may enable us to knockdown the target mRNA and thus, expecting to lower expression of protein in the cell. These proteins which are involved in lipid synthesis will be available in limited quantity to synthesize long chain fatty acids in lower quantity. Till date many knock down or knock out chickens have been developed of which most of them are involved in improving growth by lowering expression of negative regulators of growth^12,13^. However, there is no report available in any species for minimizing the cholesterol content in serum or eggs through genetic manipulation such as RNAi, which enforce permanent change at genome level and is inherited to the subsequent generation. The study on knocking down the expression of ACACA and SREBP1 genes through shRNA-based RNAi in chicken is not available in the literature. In the present study, we report for the first time to have developed transgenic chicken for reducing cholesterol content in serum and eggs.

## Results

### shRNA constructs transferred and hatching performance observed in the experiment

Two shRNA constructs for ACACA and two shRNA constructs for SREBP1 genes were transferred to the chicken through sperm mediated gene transfer method. The sperms were collected from cock’s semen and made it ready for transfection by electroporation with linear shRNA cassettes. The transfected sperms were transferred by artificial insemination to the recipient hens to obtain fertile eggs from the inseminated hens. The fertility percentage in the treatment group irrespective of gene specific shRNA groups varied from 40 to 60% while in the control group, it was 83.3% (Table 1). The hatchability percentage on fertile and total eggs set basis varied from 92.9 to 100%, and 40.0 to 56.7% in the treatment group across the genes while the estimates in the control group was 96% and 80%, respectively.

**Table 1:** Hatching performance of birds in transgenic and control groups during 1st generation.

Gene	Clone name	No. of eggs set in the hatcher	No. of fertile eggs detected upon candling	Fertility (%)	No. of chicks hatched	Hatchability on fertile egg set (%)	Hatchability on total egg set (%)	Positive transgenic chicks	Percentage of obtaining transgenic chicks (%)
ACACA	shRNA3	30	14	46.7	13	92.9	43.3	1	7.7
	shRNA4	30	17	56.7	16	94.1	53.3	1	6.3
SREBP1	shRNA1	30	12	40.0	12	100.0	40.0	1	8.3
	shRNA2	30	18	60.0	17	94.4	56.7	1	5.9
	Control group	30	25	83.3	24	96.0	80.0	0	0

Fertile eggs were incubated in the egg incubator and candling was done on 18th day to screen the fertile eggs. On 18th day, ggs were incubated in the hatcher and chicks were hatched on 21st day. Two shRNA clones each of ACACA and SREBP1 genes were transferred to the host through sperm mediated gene transfer and the transfected sperms were inseminated to the hens under treatment group while normal sperms were inseminated to the hens under control group. Fertile eggs from both treatment and control groups were collected for hatching to chicks.

### Transgenic birds are developed

**Figure 1:**
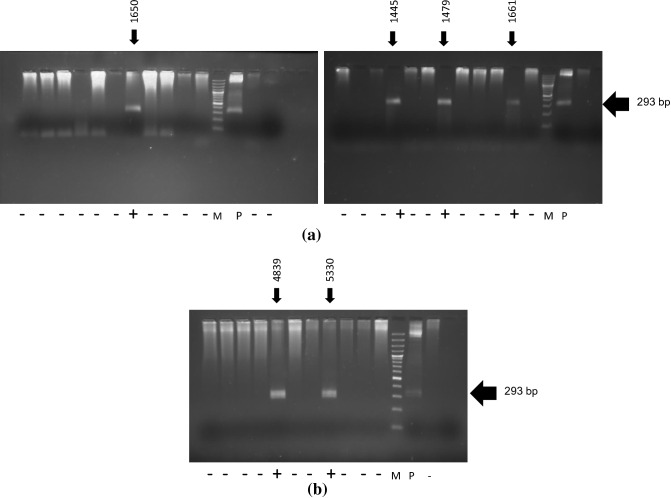
Screening of birds for identification of transgene integration during 1st and 2nd generation. ‘+’ indicates transgenic positive birds possessing 293 bp amplified product. The 293 bp fragment is located at the back bone of pENTR™/U6 vector. ‘−’ indicates transgenic negative birds. The numericals with arrow indicate the wing band No. of transgenic birds. M = 100 bp ladder DNA marker. P = Amplified fragment from shRNA recombinant plasmid as positive control. (**a**) = 1st generation birds. (**b**) = 2nd generation birds.

**Figure 2:**
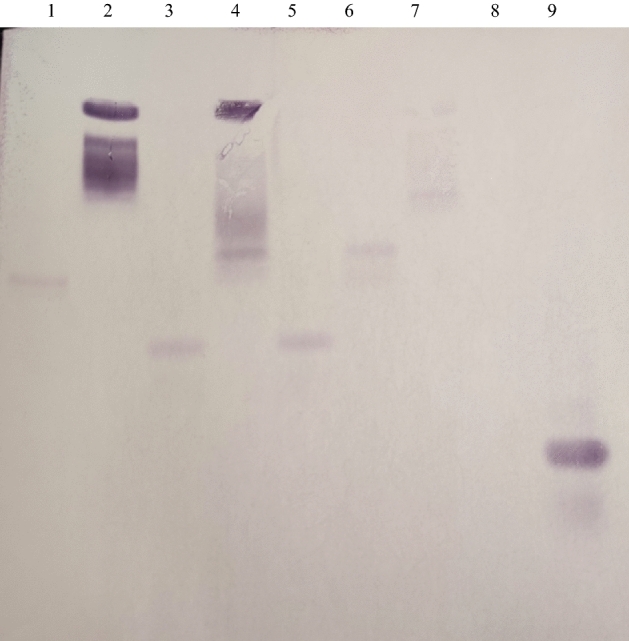
Southern blotting of transgenic birds. Lane 1: shRNA construct, Lanes 2–5: Transgenic birds of 1st generation, Lane6&7: Transgenic birds of 2nd generation; Lane8: Non-transgenic control bird; Lane9: Amplified product from shRNA plasmid construct acted as positive control for Southern blotting. Before running the gel, genomic DNA was digested with *Apa*I restriction enzyme. The digested sample was run on 0.8% agarose gel and was transferred to PVDF membrane. A 286 bp fragment located at the back bone of pENTR™/U6 vector was amplified from the shRNA construct and was used for preparation of probe. The probe was labelled with biotin-streptavidin conjugated to alkaline phosphatase and detected with NBT/BCIP for spot hybridization.

**Figure 3:**
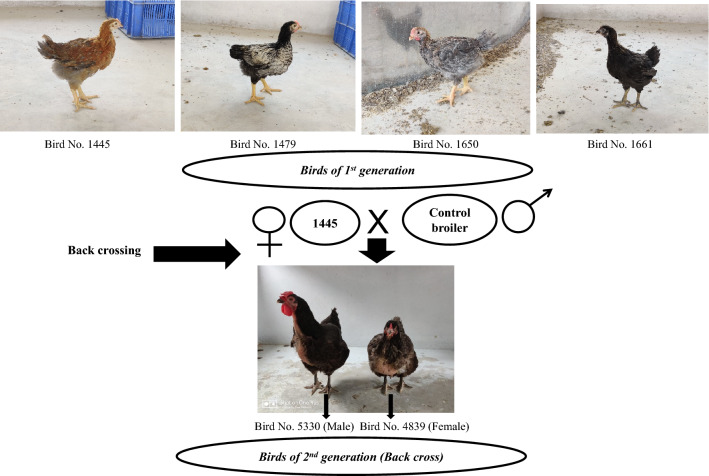
Transgenic birds developed at 1st and 2nd generation. At 1st generation, all the birds were female. At 1st generation, bird No. 1445: ACACA shRNA3; 1479: ACACA shRNA4; 1650: SREBP shRNA2 and 1661: SREBP shRNA2 were produced. Female transgenic birds of 1st generation was back crossed with control broiler birds as male parents to produce back cross transgenic progenies carrying shRNA constructs. At 2nd generation, two positive transgenic birds for ACACA shRNA construct were obtained. At 2nd generation, bird No. 4839 is male and bird No. 5330 is female.

**Figure 4:**
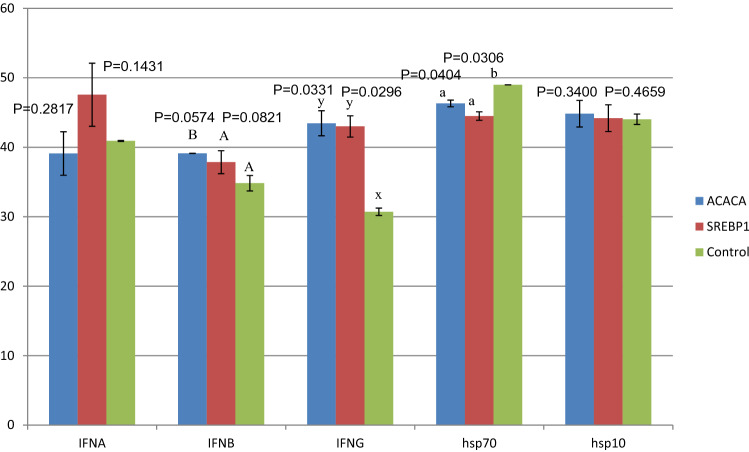
Expression of hsps (hsp10 and hsp70) and immune response (IFNA, IFNB and IFNG) genes in blood cells of ACACA and SREBP1 transgenic and control birds. Different superscripts (a/b) or (A/B) or (x/y) indicate significance at P < 0.05 for expression of hsp70, IFNB and IFNG genes, respectively. The IFNB expression between SREBP1 transgenic and control groups differed significantly. The IFNG expression between ACACA transgenic and control groups, and SREBP1 transgenic and control groups differed significantly. The hsp70 expression between ACACA transgenic and control, and SREBP1 transgenic and control groups differed significantly. The expression was checked at 6 weeks of age in birds of 1st generation.

A total of 82 chicks across the treatment and control groups were hatched. All the chicks were screened by PCR (Fig. 1a, b) and the positive chicks were re-confirmed by DNA sequencing (Supplementary Fig. 1) and Southern blotting (Fig. 2). From each treatment group, 1 positive transgenic chick was obtained and a total of 4 transgenic chicks were produced (Fig. 3). All the transgenic birds were female. The efficiency of production of transgenic birds varied from 5.9 to 8.3% across shRNA groups under both ACACA and SREBP1 genes. The expression of ACACA and SREBP1 proteins in transgenic and control birds were detected by Sandwich ELISA, which revealed 45.8 to 78.2% lower expression of ACACA and 48.1 to 74.6% lower expression of SREBP1 in the respective transgenic birds as compared to the control birds at different titres in 1st generation (Table 2a). In2nd generation also, the expression of ACACA was 46.9 to 70.6% lower across the titres in the transgenic group than the control one (Table 2b). Thus, all 4 transgenic birds showed knock down of expression of the ACACA and SREBP1 genes indicating efficiency of the shRNA molecules in silencing expression of the genes. We assessed expression of hsp70 and hsp10 genes in blood cells of transgenic and control animals. In transgenic birds, blood cells showed significantly (P = 0.0404 for ACACA and P = 0.0306 for SREBP1 transgenic groups) lower stress in terms of hsp70 expression as compared to that of the control birds under normal managemental condition indicating better cellular house-keeping function in transgenic birds. But, there were no significant differences (P = 0.34 for ACACA group and P = 0.4659 for SREBP1 group) of hsp10 expression in blood cells of transgenic and control birds. As far as immune response genes are concerned, there were significant differences of expression of interferon-alpha (IFNB) between ACACA transgenic and control groups and of interferon-gamma (IFNG) between both ACACA and SREBP1 transgenic groups with control group indicating effects of shRNA constructs integrated in the transgenic birds on immune system (Fig. 4).

**Table 2:** Detection of ACACA and SREBP1 proteins (OD_650_ values) in the serum of transgenic and control birds at the age of 6 weeks by sandwich ELISA.

	Titre (OD_650_) (Mean)								
	1:10	1:100	1:250	1:500	1:1000	1:1500	1:2000	1:4000	1:8000
(**a**) **Groups**									
Control group (ACACA)	0.467	0.434	0.403	0.361	0.322	0.286	0.252	0.230	0.198
ACACA transgenic group	0.253	0.205	0.177	0.168	0.150	0.140	0.130	0.093	0.043
P	0.0005	0.0014	0.0120	0.0320	0.0360	0.0246	0.0059	0.0286	0.0069
Percentage of reduction of ACACA protein content in serum of ACACA transgenic birds (%)	45.8	52.7	56.0	53.5	53.4	51.0	48.4	59.5	78.2
Control group (SREBP1)	0.469	0.439	0.411	0.389	0.345	0.300	0.261	0.225	0.193
SREBP1 transgenic group	0.243	0.205	0.178	0.165	0.152	0.140	0.125	0.098	0.049
P	0.00008	0.0127	0.0028	0.0529	0.0067	0.0044	0.0104	0.0003	0.0108
Percentage of reduction of SREBP1 protein content in serum of SREBP1 transgenic birds (%)	48.1	53.3	56.6	57.5	55.9	53.3	52.1	56.4	74.6
(**b**) **Groups**									
Control group (ACACA)	0.469	0.427	0.398	0.376	0.347	0.289	0.247	0.202	0.174
ACACA transgenic group	0.247^A^	0.209	0.177	0.168	0.157 ^A^	0.143	0.131	0.095	0.051
P	0.0461	0.0241	0.0335	0.0278	0.0280	0.0362	0.0247	0.0204	0.0362
Percentage of reduction of ACACA protein content in serum of ACACA transgenic birds	89.8	51.0	55.5	55.3	54.7	50.5	46.9	52.9	70.6

**a** Birds of 1st generation. **b** Birds of 2nd generation. Controls (ACACA) in both (a) and (b) indicate quantification of ACACA protein in serum of the control birds. Control (SREBP1) indicates quantification of SREBP1 protein in terms of OD_650_ values in serum of control birds during 1st generation (a). Comparisons of quantity of ACACA protein in serum between control (ACACA) and ACACA knockdown birds of 1st (a) and 2nd (b) generations reveal highly significant differences of this protein between two groups in each generation demarcated by different superscripts (a/b) and (A/B), respectively. Comparison of quantity of SREBP1 protein in serum between control (SREBP1) and SREBP1 knockdown birds of 1st generation (a) reveals highly significant differences of this protein between these two groups demarcated by different superscripts (x/y). Both ACACA and SREBP1 proteins in serum of knock down birds of ACACA and SREBP1 groups were lower than those of control birds indicating efficiency of silencing expression of the SCACA and SREBP1 genes in knock down birds.

### Growth performance in transgenic birds varied from control birds

The body weights of transgenic and control birds were recorded at different ages. The body weights were significantly differed between transgenic and control birds at day-old, 5, 6, 8 and 10 weeks of age except day old body weight in SREBP1 transgenic group (Table 3). During adult stage (20 weeks and beyond), body weight did not differ significantly between ACACA and SREBP1 transgenic with control group of birds. However, the growth pattern reveals that as age progressed (20 weeks onward) body weight did not differ significantly between transgenic and control groups.

**Table 3:** Growth performance (Mean ± standard deviation) of female transgenic and control birds during 1st generation.

Groups	Body weight at Day old age (g)	Body weight at 5 weeks (g)	Body weight at 6 weeks (g)	Body weight at 8 weeks (g)	Body weight at 10 weeks (g)	Body weight at 20 weeks (g)	Body weight at 32 weeks (g)	Body weight at 40 weeks (g)	Body weight at 52 weeks (g)
Control group (n = 2)	37.1 ± 2.9 ^a^	1008.7 ± 57.6 ^c^	1191.5 ± 57.7 ^b^	1504.9 ± 50.3 ^b^	1649.0 ± 52.3 ^b^	2583.0 ± 119.1	3144.0 ± 96.0	3827.0 ± 68.0	4450.0 ± 104.0
ACACA transgenic birds (n = 2)	43.7 ± 2.7 ^b^	666.0 ± 55.3 ^a^	871.7 ± 101.2 ^a^	1138.0 ± 65.7 ^a^	1293.2 ± 67.1 ^a^	2498.0 ± 259.0	3237.0 ± 137.0	3817.0 ± 35.0	4757.0 ± 106.0
P	0.0501	0.0150	0.0269	0.0298	0.0329	0.3663	0.2575	0.4264	0.0673
Percent change in ACACA transgenic group (%)	17.8	− 33.9	− 26.8	− 24.3	− 21.5	− 3.2	2.9	− 0.2	6.8
SREBP1 transgenic birds (n = 2)	37.0 ± 0.8 ^a^	766.2 ± 46.7 ^a^	981.0 ± 48.6 ^a^	1239.5 ± 44.9 ^a^	1350.0 ± 48.8 ^a^	2306.0 ± 123.0	3285.0 ± 124.0	3946.0 ± 62.0	4671.0 ± 140.0
P	0.4851	0.0286	0.0416	0.0186	0.0159	0.0742	0.1650	0.0739	0.1166
Percent change in SREBP1 transgenic group (%)	− 0.2	− 24.0	− 17.6	− 17.6	− 18.1	− 10.7	4.5	3.1	4.9

Different superscripts (column-wise) indicate significant differences. Body weights of all the experimental birds were measured in the electronic weighing balance at day old, 5, 6, 8, 10, 20, 32, 40 and 52 week of age. Body weights were compared between ACACA transgenic and control group, and SREBP1 transgenic and control group to determine the effect of knock down on body weights in transgenic chicken.

### Blood parameters varied between transgenic and control birds

Different blood parameters such as haemoglobin% (Hb%), RBC count, packed cell volume (PCV), erythrocyte sedimentation rate (ESR) and mean corpuscular haemoglobin (MCH) were estimated in both transgenic and control birds at the age of 26 weeks (Table 4). Of all these parameters, only ESR at 1st hour varied significantly (P = 0.0432 for ACACA group and P = 0.0499 for SREBP1 group) between control and transgenic birds where ESR was increased by 115.3% in ACACA transgenic birds and by 69.2% in SREBP1 transgenic birds over the control ones.

**Table 4:** Blood parameters (Mean ± standard deviation) in female transgenic and control birds at 26 weeks of age during 1st generation.

Groups	Hb% (g%)	RBC (Million/Cumm)	PCV (%)	ESR (1st hour)	MCH (pg)
Control group (n = 2)	13.5 ± 0.2	2.8 ± 0.06	41.3 ± 0.6	3.25 ± 0.6 ^a^	46.6 ± 0.6
ACACA transgenic birds (n = 2)	12.8 ± 0.1	2.8 ± 0.04	39.9 ± 1.2	7.0 ± 0.8 ^c^	44.3 ± 0.3
P	0.0667		0.2650	0.0432	0.0699
Percent change in ACACA transgenic group (%)	− 5.2	0	− 3.4	115.3	− 4.9
SREBP1 transgenic birds (n = 2)	13.4 ± 0.4	2.8 ± 0.04	41.7 ± 0.5	5.5 ± 1.2 ^b^	45.9 ± 0.9
P	0.4464	0.3856	0.3733	0.0499	0.3172
Percent change in SREBP1 transgenic group (%)	− 0.7	0	0.9	69.2	− 1.5

Different superscripts (column-wise) indicate significant differences. The RBC indices viz. RBC count, Haemoglobin (Hb)%, packed cell volume (PCV), erythrocyte sedimentation rate (ESR) and mean corpuscular haemoglobin (MCH) were measured in transgenic as well as control birds to compare between transgenic and control groups.

### Differential count in transgenic vs control birds

The total WBC count did not differ significantly (P = 0.0699) between transgenic and control group of birds (Table 5). But, eosinophil and monocyte percentage varied significantly (P = 0.0076 in ACACA group and P = 0.0349 in SREBP1 group) between transgenic and control groups of birds. The eosinophil percent increased by 76.4% in ACACA transgenic birds and decreased by 11.7% in SREBP1 transgenic birds as compared to the control birds. However, percentage of other white blood cells did not differ significantly between transgenic and control groups.

**Table 5:** Different blood cells (Mean ± standard deviation) in female transgenic and control birds at 26 weeks of age during 1st generation.

Groups	Total WBC (cells/cumm)	Neutrophils (%)	Lymphocytes (%)	Eosinophils (%)	Monocytes (%)	Basophils (%)
Control group (n = 2)	2067.0 ± 27.0	52.7 ± 2.2	43.2 ± 2.9	1.7 ± 0.2^b^	2.7 ± 0.4	0
ACACA transgenic group (n = 2)	1940.0 ± 16.0	55.0 ± 4.1	40.0 ± 4.1	3.0 ± 0 ^c^	2.0 ± 0	0
P	0.0699	0.3763	0.3156	0.0076	0.1577	
Percent change in ACACA transgenic group (%)	− 6.1	4.3	− 7.4	76.4	− 25.9	0
SREBP1 transgenic group (n = 2)	1910.0	57.0 ± 2.4	39.5 ± 2.9	1.5 ± 0.4 ^a^	2.0 ± 0	0
P	0.0652	0.1884	0.2487	0.0349	0.1577	
Percent change in SREBP1 transgenic group (%)	− 7.5	8.1	− 8.5	− 11.7	− 25.9	0

Different superscripts (column-wise) indicate significant differences at P < 0.05. WBCs were counted in blood of transgenic as well as control birds at 26 weeks of age to compare between the groups.

### Serum cholesterol and triglycerides reduced in transgenic birds

The serum total cholesterol content was reduced by 21.2 and 23.8% in ACACA and SREBP1 transgenic birds, respectively during 1st generation (Table 6). The serum LDL cholesterol content was reduced by 36.7 and 20.9% in ACACA and SREBP1 transgenic birds, respectively. The serum HDL content was significantly increased by 12.6% in ACACA transgenic group, but decreased by 26.6% in SREBP1 transgenic group over the control ones. The serum triglyceride contents were also reduced by 10.5 and 35.6% in ACACA and SREBP1 transgenic birds, respectively over the control group of birds.

**Table 6:** Serum biochemical parameters (Mean ± standard deviation) such as blood urea, creatinine, uric acid, albumin, cholesterol, triglycerides, HDL and LDL contents in female transgenic (n = 2 for ACACA transgenic group and n = 2 for SREBP1 transgenic group) and control birds (n = 2) at 26 weeks of age during 1st generation.

Groups	Blood Urea (mg%)	Serum creatinine (mg%)	Serum Uric acid (mg%)	Serum albumin (mg%)	Total cholesterol (mg/dl)	Triglycerides (mg/dl)	HDL (mg/dl)	LDL (mg/dl)
Control group	12.70 ± 1.10	0.47 ± 0.04	1.90 ± 0.09 ^b^	3.50 ± 0.10	116.80 ± 7.60^b^	88.40 ± 14.40 ^b^	61.80 ± 2.2 ^b^	30.50 ± 4.30 ^b^
ACACA transgenic group	13.00 ± 0.80	0.45 ± 0.04	1.60 ± 0.04 ^a^	3.70 ± 0	92.00 ± 9.50	79.10 ± 6.1	69.60 ± 13.7	19.30 ± 2.60
P	0.4388	0.3709	0.0310	0.1095	0.0507	0.0523	0.0536	0.0560
Percent change in ACACA transgenic group (%)	2.30	− 4.20	− 15.70	5.70	− 21.20	− 10.50	12.60	− 36.70
SREBP1 transgenic group	12.00 ± 2.40	0.45 ± 0.04	1.40 ± 0.40 ^a^	3.50 ± 0	88.90 ± 2.20 ^a^	56.90 ± 1.90^a^	45.30 ± 22.80 ^a^	24.10 ± 11.80 ^a^
P	0.4266	0.3709	0.0245		0.0130	0.0218	0.0328	0.0370
Percent change in SREBP1 transgenic group (%)	− 5.50	− 4.20	− 26.30	0	− 23.80	− 35.60	− 26.60	− 20.90

Different superscripts (column-wise) indicate significant differences. The serum biochemical parameters, which are indicators of overall health status of birds were compared between transgenic and control groups. The serum cholesterol, triglycerides and LDL contents were significantly reduced in transgenic groups as compared to the control group on account of silencing ACACA and SREBP1 genes in chicken.

### Serum biochemical parameters varied between transgenic and control groups

Different biochemical parameters such as blood urea, serum creatinine, serum uric acid and serum albumin contents were estimated in transgenic as well as control birds in which serum uric acid differed significantly (P = 0.0310 for ACACA group and P = 0.0245 for SREBP1 group) between transgenic and control groups (Table 6). In ACACA and SREBP1 transgenic groups, uric acid content was reduced by 15.7 and 26.3%, respectively over the control group of birds.

### Serum progesterone and estrogen estimated in transgenic birds

Progesterone and estrogen content in the serum of transgenic and control birds were estimated at 51 weeks of age in 1st generation and 25 weeks of age in 2nd generation. The progesterone content was significantly (P = 0.0608 for ACACA group and P = 0.0207 for SREBP1 group) higher in transgenic groups as compared to the control group during 1st generation (Table 7). The transgenic birds had 278.2 and 165.2% higher progesterone content in ACACA and SREBP1 groups, respectively as compared to the control birds. The ACACA and SREBP1 transgenic birds showed non-significant differences at P = 0.2414 and P = 0.4152, respectively in serum estrogen content as compared to the control birds.

**Table 7:** Progesterone and estrogen content in serum of adult transgenic and control birds.

Wing Band No of birds	Individual bird-wise progesterone and estrogen estimates							Overall transgenic and control group-wise progesterone and estrogen estimates and difference between transgenic and control groups during 1st generation		
	Molecule	Age (Weeks)	Generation	Ovulation cycle	Gender	Progesterone (ng/ml)	Estrogen (pg/ml)	Groups	Progesterone (ng/ml)	Estrogen (pg/ml)
First generation										
1479	ACACA_shRNA4	51	1st	1–2 h after ovulation	Female	0.98	209.50	Control group	0.23 ± 0.07 ^a^	204.40 ± 21.50
1445	ACACA_shRNA3	51	1st	1–2 h after ovulation	Female	0.76	286.40	ACACA transgenic group	0.87 ± 0.15 ^c^	247.90 ± 54.30
								P	0.0508	0.2414
1650	SREBP1_shRNA2	51	1st	1–2 h after ovulation	Female	0.55	182.80	Percent change in ACACA transgenic group (%)	278.20	21.20
1661	SREBP1_shRNA2	51	1st	1–2 h after ovulation	Female	0.66	244.70	SREBP1 transgenic group	0.61 ± 0.07 ^b^	213.70 ± 43.70
								P	0.0207	0.4152
1721	Control	51	1st	1–2 h after ovulation	Female	0.18	189.20	Percent change in SREBP1 transgenic group (%)	165.20	4.50
1728	Control	51	1st	1–2 h after ovulation	Female	0.28	219.70			
Second generation										
5330	ACACA_shRNA3	25	2nd	-	Male	0.16	86.26			
4839	ACACA_shRNA3	25	2nd	1–2 h after ovulation	Female	0.44	138.16			
5333	Control	25	2nd	-	Male	0.25	108.55			

The hormones were estimated during 1–2 h after ovulation at 51 weeks of age by chemiluminescence immunoassay to compare between transgenic and control groups.

### Egg production and quality traits varied between transgenic and control birds

Egg production upto 52 weeks in the birds of SREBP1 transgenic group was significantly (P = 0.0583) reduced by 37.8% over the control birds (Table 8). But, in case of ACACA transgenic group, there was no significant difference (P = 0.2915) of egg production compared to the control group. However, in both the cases, the egg weight did not vary significantly between transgenic and control groups (Table 9). The age at sexual maturity did not vary significantly between transgenic and control groups. The yolk%, yolk index and shell% varied significantly between transgenic and control groups. In case of yolk%, transgenic birds for SREBP1 showed 20.6% lower magnitude than the birds of control group. But, ACACA transgenic birds did not have significant difference (P = 0.1853) of yolk% with control group. In case of yolk colour index, ACACA transgenic birds showed 22.2% higher index than the control birds while there was no significant difference between SREBP1 transgenic and control birds. The egg shell% was significantly higher in both ACACA (P = 0.0500) and SREBP1 (P = 0.0381) transgenic birds where SREBP1 birds had 35.8% higher shell% than the control birds.

**Table 8:** Egg production (Mean ± standard deviation) upto 52 weeks and egg quality traits (Mean ± standard deviation) at 52 weeks of age in transgenic (n = 2 for ACACA transgenic group and n = 2 for SREBP1 transgenic group) and control birds (n = 2) during 1st generation.

Groups	Egg production upto 52 weeks (No.)	Age at sexual maturity (Days)	Albumin %	Yolk%	Shell%	Haugh unit	Yolk colour index
Control group	82.0 ± 11.0	178.7 ± 9.2	55.7 ± 2.4	34.9 ± 1.7 ^b^	9.2 ± 0.9 ^a^	84.0 ± 2.1	9.0 ± 0.1
ACACA transgenic group	77.0 ± 9.0	189.1 ± 12.4	51.8 ± 1.9	36.3 ± 2.0 ^b^	11.8 ± 1.0 ^b^	91.0 ± 1.8	11.0 ± 0.1
P	0.2915	0.0627	0.2381	0.1853	0.0500	0.4813	0.0588
Percent change in ACACA transgenic group (%)	− 6.1	5.8	− 7.0	4.0	28.2	8.3	22.2
SREBP1 transgenic group	51.0 ± 11.0	192.6 ± 16.3	59.7 ± 2.1	27.7 ± 1.7 ^a^	12.5 ± 1.0 ^b^	87.0 ± 1.7	8.0 ± 1.0 ^a^
P	0.0583	0.0649	0.4162	0.0436	0.0381	0.2901	0.2783
Percent change in SREBP1 transgenic group (%)	− 37.8	7.7	7.1	− 20.6	35.8	3.5	− 11.1

Different superscripts (column-wise) indicate significant differences at P < 0.05. The internal egg quality traits viz. albumin%, yolk%, shell%, Haugh unit and yolk colour index at 52 weeks of age were measured in egg quality testing machine in both transgenic and control groups. Yolk% and shell% of eggs between transgenic and control groups differed significantly.

**Table 9:** Egg cholesterol content (Mean ± standard deviation) in transgenic (n = 2 for ACACA transgenic group and n = 2 for SREBP1 transgenic group) and control broiler birds (n = 2) at 52 weeks of age during 1st generation.

	Egg wt (g)	Total cholesterol (mg/100 g of egg)	LDL (mg/100 g of egg)
Control group	60.0 ± 1.8	509.3 ± 27.1	432.6 ± 11.1
ACACA transgenic group	55.0 ± 1.3	436.1 ± 33.0	387.5 ± 20.9
P	0.2263	0.0577	0.0574
Percent change in ACACA transgenic group (%)	− 8.3	− 14.3	− 10.4
SREBP1 transgenic group	62.07 ± 1.6	441.7 ± 33.4	373.2 ± 46.5
P	0.4020	0.0579	0.0522
Percent change in SREBP1 transgenic group (%)	3.4	13.2	13.7

The total cholesterol and LDL content in eggs were measured in knock down and control groups.

### Egg cholesterol reduced in transgenic birds

We analysed cholesterol content in eggs of transgenic and control birds at the age of 52 weeks. The cholesterol content was numerically lower in both ACACA (P = 0.0577) and SREBP1 (P = 0.0579) transgenic birds by 14.3 and 13.2%, respectively over the control birds (Table 9). The egg LDL content was also numerically lower in ACACA (P = 0.0574) and SREBP1 (P = 0.0522) transgenic birds by 10.4 and 13.7%, respectively as compared to the control birds.

### Egg mineral contents varied between transgenic and control birds

We estimated important minerals viz. Cu, Fe, Zn, Mn, Mg, Cr, Ni, Ca, B and Se in eggs of transgenic and control birds at 52 weeks of age (Table 10). We observed significant differences of Cu, Zn, Mn, and B between the eggs of transgenic and control birds. The Zn, Mn and B contents were higher in eggs of ACACA and SREBP1 transgenic birds while Cu content was significantly lower in transgenic birds as compared to the control ones.

**Table 10:** Mineral contents (Mean ± standard deviation) in eggs of transgenic (n = 2 for ACACA transgenic group and n = 2 for SREBP1 transgenic group) and control birds (n = 2) at 52 weeks of age during 1st generation.

	Egg wt	Cu	Fe	Zn	Mn	Mg	Cr	Ni	Ca	B	Se
Control group	50.4 ± 5.2 ^a^	0.065 ± 0.057 ^b^	1.79 ± 0.24	0.799 ± 0.015^a^	0.024 ± 0.002 ^a^	8.12 ± 0.40	0.046 ± 0.03	0.008 ± 0.001	29.3 ± 1.4	0.022 ± 0.02 ^a^	0.009 ± 0.01^a^
ACACA transgenic group	60.5 ± 1.5 ^b^	0.030 ± 0.010 ^a^	6.90 ± 22.08	0.945 ± 0.087^b^	0.041 ± 0.044 ^b^	7.58 ± 0.68	0.036 ± 0.05	0.007 ± 0.003	31.3 ± 4.8	0.046 ± 0.09 ^b^	0.020 ± 0.01
P	0.0056	0.0306	0.0514	0.0238	0.0358	0.0810	0.2895	0.0782	0.2510	0.0281	0.0579
Percent change in ACACA transgenic group (%)	20.0	− 53.8	285.4	18.2	70.8	− 6.6	− 21.7	− 12.5	6.8	109.0	122.2
SREBP1 transgenic group	52.3 ± 1.1 ^a^	0.033 ± 0.012 ^a^	6.64 ± 37.06	0.945 ± 0.081^b^	0.055 ± 0.070 ^b^	7.35 ± 0.73	0.053 ± 0.1	0.009 ± 0.003	35.7 ± 4.1	0.045 ± 0.06 ^b^	0.013 ± 0.009 ^b^
P	0.2159	0.0435	0.0519	0.0009	0.0232	0.1431	0.4761	0.3478	0.0580	0.0236	0.0328
Percent change in SREBP1 transgenic group (%)	3.7	− 49.2	270.9	18.2	129.1	− 9.4	15.2	12.5	21.8	104.5	44.4

Mineral contents viz. Cu, Fe, Zn, Mn, Mg, Cr, Ni, Ca, B and Se in eggs of both knock down and control groups were measured in iCAP7200 ICP OES Duo analyzer. Different superscripts (column-wise) indicate significant differences. The mineral contents such as Cu, Zn, Mn and B in eggs differed significantly between transgenic and control groups.

### Growth, blood and biochemical parameters validated in the transgenic birds produced in 2nd generation

All the transgenic birds of 1st generation were of female, which were used further as female parents for back crossing with control broiler birds as male parents to produce back cross progenies to examine inheritance of the shRNA construct. The hatching schedule of the birds has described in Table 11. In back cross, two positive transgenic chicks for ACACA gene were hatched (Figs. 2, 3). But, in SREBP1 group, we did not have any positive transgenic chicks. Of two positive chicks, one was of male and another one was of female, but both carried shRNA3 ACACA construct. We also compared expression of ACACA protein in the serum upto 1:8000 dilution between the transgenic and control birds through sandwich ELISA in serum samples and found lower expression of ACACA in serum of ACACA transgenic birds at 2nd generation (Table 2b). The growth performance of the birds has been mentioned in Table 12. The body weights between transgenic and control birds significantly differed at 2 (P = 0.0279), 8 (P = 0.0348) and 10 (P = 0.0303) weeks of age. At 2nd week, body weights of transgenic birds were reduced by 16.6% over the control birds while 8 weeks onward, body weight of transgenic birds increased gradually by 26 and 27.5%, respectively upto 10 weeks. In the back cross generation, we also analysed several blood parameters including Hb%, RBC count, PCV%, MCV%, MCH and MCHC% to validate the effect of shRNA on these parameters in transgenic birds. We observed significant differences of Hb% (P = 0.0188), RBC count (P = 0.0130) and ESR (P = 0.0146) between transgenic and control birds. The Hb%, RBC count and ESR was 19.5, 23.0 and 22.2% lower, respectively in transgenic birds compared to the control ones (Table 12). We also found significant difference (P = 0.0174) in platelets count which was 29.7% lower in transgenic birds than the control birds. We analysed serum cholesterol, triglycerides, HDL and LDL contents in transgenic and control birds. We found significant deifference (P = 0.0182) (reduced by 18.5%) in serum cholesterol content between ACACA transgenic and control birds. In addition, we estimated blood urea, serum creatinine, serum uric acid and serum albumin in transgenic and control birds. We found significantly (P = 0.0054) higher serum creatinine (330%) and serum uric acid (33.3%) in transgenic birds whereas blood urea and serum albumin did not differ significantly between transgenic and control group. We also estimated progesterone and estrogen content at 25 weeks of age in serum of both transgenic and control birds. The serum progesterone content in male and female transgenic, and control birds of 2nd generation were 0.16, 0.44 and 0.25 ng/ml, respectively. The serum estrogen content in male and female transgenic, and control birds were 86.2, 138.1 and 108.5 pg/ml, respectively.

**Table 11:** Hatching performance of transgenic birds in 2nd generation.

Gene	Clone name	Dam no. used for back crossing	No. of eggs set in the hatcher	No. of fertile eggs detected upon candling	Fertility (%)	No. of chicks hatched	Hatchability on fertile egg set (%)	Hatchability on total egg set (%)	Knock-down positive chicks	Percentage of obtaining positive chicks (%)
ACACA	shRNA3	1479	6	5	83.3	3	60.0	50.0	2	66.7
	shRNA4	1445	10	8	80.0	5	62.5	50.0	0	0
SREBP1	shRNA1	1650	3	2	66.7	1	50.0	33.3	0	0
	shRNA2	1661	5	4	80.0	2	50.0	40.0	0	0

Female transgenic birds of first generation was back crossed with control broiler birds as male parents to produce back cross transgenic progenies carrying shRNA constructs. Two positive transgenic chicks were hatched only for ACACA shRNA group. The efficiency of production of transgenic chicks through backcross in ACACA group is very high (66.6%).

**Table 12:** Growth performances (a), RBC parameters (b), differential count (c), serum lipids (d) and serum biochemical parameters (e) (Mean ± standard deviation) in transgenic (Values calculated from both 1 male and 1 female birds combinedly) and control birds (Values are on the basis of both 1 male and 1 female birds combinedly) in 2nd generation.

(a) Groups	Body weight at Day old age (g)	Body weight at 2 weeks (g)	Body weight at 4 weeks (g)	Body weight at 6 weeks (g)	Body weight at 8 weeks (g)	Body weight at 10 weeks (g)	Body weight at 20 weeks (g)
Control group	37.5 ± 1.4	149.0 ± 49.6 ^b^	457.0 ± 222.5	820.0 ± 315.1	1039.6 ± 370.4 ^a^	1332.0 ± 390.0 ^a^	3064.0 ± 211.0
ACACA transgenic group	34.2 ± 3.8	124.2 ± 45.5 ^a^	450.0 ± 31.1	886.6 ± 304.9	1310.7 ± 578.7 ^b^	1698.5 ± 688.0 ^b^	3113.0 ± 485.0
P	0.2333	0.0279	0.4775	0.4000	0.0348	0.0303	0.4097
Percent change in ACACA transgenic group (%)	− 8.8	− 16.6	− 1.5	8.1	26.0	27.5	1.5

(b) Groups	Hb (gm%)	RBC (Million/Cumm)	PCV (%)	MCV (%)	MCH (pg)	MCHC (%)	ESR (1st hour)
Control group	16.9 ± 1.0 ^b^	3.9 ± 0.1 ^b^	49.1 ± 1.6	127.7 ± 6.2	43.8 ± 3.3	34.3 ± 0.8	4.5 ± 0.4^b^
ACACA transgenic group	13.6 ± 3.0 ^a^	3.0 ± 0.6 ^a^	39.0 ± 8.6	127.9 ± 0	44.5 ± 1.5	34.8 ± 0.1	3.5 ± 0.6 ^a^
P	0.0188	0.0130	0.0517	0.4891	0.4082	0.2643	0.0146
Percent change in ACACA transgenic group (%)	19.5	23.0	− 20.6	0.1	1.5	1.4	− 22.2

(c) Groups	Total WBC (cells/cumm)	Neutrophils (%)	Eosinophils (%)	Basophils (%)	Lymphocytes (%)	Monocytes (%)	Platelets (cells/cumm)
Control group	2503.0 ± 33.0	59.0 ± 1.4	3.0	0	36.0 ± 1.4	2	18,500.0 ± 2121.0^b^
ACACA transgenic group	2220.0 ± 27.0	63.0 ± 0.7	3.0	0	32.0 ± 0.7	2	13,000.0 ± 4242.0^a^
P	0.1890	0.0775			0.0775		0.0174
Percent change in ACACA transgenic group (%)	− 11.3	6.7	0	0	− 11.1	0	− 29.7

(d) Groups	Serum cholesterol (mg/dl)	Triglycerides (mg/dl)	HDL (mg/dl)	LDL (mg/dl)			
Control group	127.0 ± 5.2 ^b^	104.7 ± 23.4 ^b^	66.3 ± 14.4	32.4 ± 0.8			
ACACA transgenic group	103.5 ± 20.9 ^a^	82.6 ± 54.5 ^a^	75.7 ± 11.4	33.6 ± 31.9			
P	0.0182	0.0348	0.2720	0.4823			
Percent change in ACACA transgenic group (%)	− 18.5	− 21.1	14.1	3.7			

(e) Groups	Blood Urea (mg%)	Serum creatinine (mg%)	Serum Uric acid (mg%)	Serum albumin (mg%)			
Control group	12.0 ± 4.2	0.3 ± 0.1^a^	0.8 ± 0.2^a^	3.0 ± 0.1			
ACACA transgenic group	12.0 ± 1.4	1.0 ± 0.1^b^	1.0 ± 0.1^b^	3.0 ± 0.1			
Percent change in ACACA transgenic group (%)	–	300.0	33.3	-			
P		0.0054	0.0212				

Different superscripts (column-wise) indicate significant differences. Significant differences of traits were observed between control and transgenic birds for body weights at 2, 8 and 10 weeks of age; Haemoglobin (Hb) and RBC count; platelets count; serum cholesterol, triglycerides, creatinine and uric acid.

### Semen quality varied between transgenic and control birds

**Figure 5:**
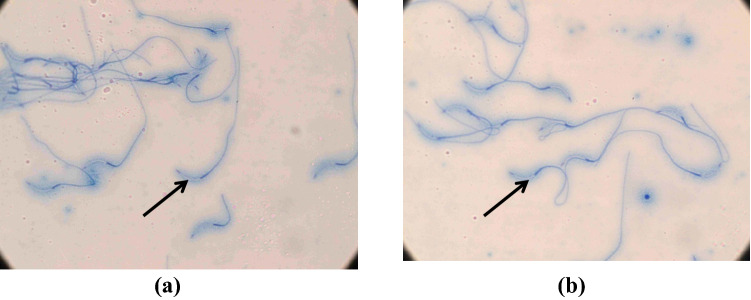
Acrosomal integrity of sperm of ACACA shRNA transgenic (**a**) and control (**b**) males. Acrosomal integrity expressed in terms of sperms are viewed under microscope with 20× magnification.

In the 2nd generation, we obtained one ACACA transgenic male bird. We compared semen quality parameters in the transgenic male and control male birds (Table 13, Supplementary Figs. 2, 3). The semen volume was higher by 81.25% in transgenic bird compared to the control bird. Likewise, sperm concentration was also higher by 60.86% in transgenic bird. But, the individual motility of sperm was lower in transgenic bird compared to the control one. There was no difference of abnormal sperm concentration and acrosomal integrity of the sperm between transgenic and control bird (Fig. 5). But, MTT dye reduction by sperm was higher by 24.62% in control bird as compared to the transgenic bird indicating better fertilizing ability of sperms.

**Table 13:** Semen quality parameters in transgenic and control male birds.

Wing Band No	Age	Volume (ml)	Concentration (Million/ul)	Individual motility (%)	Live sperm (%)	Abnormal sperm (%)	Acrosomal integrity (%)	MTT (nm formazan/min/million sperm)
5333 (Control)	30 weeks	0.3	2.3	80.0	77.0	2.0	99.0	6.1
5330 (ACACA transgenic)	30 weeks	0.6	3.7	75.0	82.0	2.0	99.0	4.6
Percent change in ACACA transgenic bird (%)		81.2	60.9	6.3	6.5	0	0	24.6

The semen quality was assessed in cocks at 30 weeks of age. Semen volume and sperm concentrations were higher in transgenic male than those in control male. MTT dye reduction by sperm was higher in control bird as compared to the transgenic bird indicating better fertilizing ability of sperms of control birds.

## Discussion

A total of 4 transgenic female birds were produced where two birds pertained to ACACA shRNAs and two birds were associated with SREBP1 shRNAs. The transgenic birds were developed through SMGT method. Though efficiency for production of transgenic birds was upto 8%, it is considered as quite better in case of poultry compared to the large livestock species. However, all these four birds were backcrossed with a normal male bird and we produced one transgenic male and one transgenic female birds associated with ACACA shRNA3. Through backcrossing, we confirmed the inheritance of shRNA recombinant molecule from parent to offspring generation. We suggest that through SMGT method, we can develop transgenic birds and can be reproduced through subsequent breeding. We also observed lower level of ACACA protein in ACACA transgenic birds of this generation compared to the control bird. It may be due to knock down of ACACA gene expression in the transgenic birds on account of functionality of ACACA RNAi construct integrated in the transgenic birds.

In the founder generation, body weight varied significantly between ACACA and SREBP1 transgenic and control birds upto 10 weeks of age and beyond that, there were no significant differences between transgenic and control groups. But, the growth pattern was different in first and second generation. During first generation, growth of ACACA transgenic birds was lower than that of control birds while in second generation, body weight of ACACA transgenic birds were higher than control ones at 6 weeks of age and onwards. However, in first generation, there was no significant difference of body weights during 20 weeks onward. In second generation also, we observed similar trend during adult stage. Such trends of body weights in transgenic and control birds indicate that there might not be significant impact of knock down of ACACA and SREBP1 genes on body weight during adult stage.

Several blood parameters which are indicative of health of animals were assessed in transgenic and control birds. We observed non-significant differences of Hb%, RBC count, PCV% and MCH between transgenic and control birds, but there was significant difference of ESR between them in 1st and 2nd generation. But, during 2nd generation, we noticed significant differences of Hb% while PCV, MCV, MCH and MCHC did not differ significantly between transgenic and control birds. During 2nd generation, ESR was lower in transgenic birds than the control ones, which may be due to lower RBC count in transgenic birds. It is known that ESR is not a specific diagnostic test of inflammation but may be elevated in acute or chronic inflammatory conditions or pathological conditions. The higher ESR occurs on account of presence of C-reactive protein or fibrinogen in blood of animals. However, apparently, we have not noticed any inflammation in the transgenic birds of 1st generation and control birds of 2nd generation, and they were healthy having good vigour. Thus, it may be inconclusive that knock-down of ACACA/SREBP1 has any association with ESR. In case of Hb%, the lower magnitude in transgenic birds may be due to lower RBC count than that of control birds. But, the total WBC count did not differ between transgenic and non-transgenic birds. However, differential count revealed that the eosinophil% differed between two groups of birds in 1st generation, but not in 2nd generation. The better body weights of transgenic birds during 2nd generation made the WBC and differential counts normal in transgenic birds, which were similar to the control group. Immunologically, WBC is extremely important as they provide immunity to the birds against infectious microbes. However, eosinophil number was higher in transgenic birds reflecting potential of the birds to combat parasitic infection or allergic reaction in the body.

To determine the stress of the cells of transgenic and control birds, we analysed heat shock proteins in blood cells. Heat shock proteins (Hsps) are among the most conserved proteins expressed in the cells normally for house-keeping functions. They are over-expressed when exposed to a stress. They act as intracellular chaperones, which correct unfolding or misfolding proteins so to keep cell with normal function. Of all hsps, the hsp10 and hsp70 play immense role in the body to protect cells from physical as well as physiological stress. Hsp10 functions as chaperone in mitochondria and is associated with a variety of activities in immunomodulation and cell proliferation and differentiation^16–19^. The Hsp70 functions in a wide range of cellular house-keeping activities including the folding of newly synthesized proteins, the translocation of polypeptides into mitochondria, and the endoplasmic reticulum, refolding of misfolded denatured proteins, solubilizing aggregated proteins and help in cellular degradation machineries to clear aberrant proteins and protein aggregates^20^. Thus, it protects cells from the deleterious effects of proteotoxic stresses and pathophysiological conditions^21,22^. In our study, in ACACA transgenic birds, blood cells showed lower stress in terms of hsp70 expression as compared to that of the control birds under normal managemental condition. But, there were no significant differences of stress of blood cells in transgenic and control birds with respect to hsp10 expression.

We also assessed the expression of IFN-alpha, IFN-beta and IFN-gamma genes in blood cells of both transgenic and control birds. Interferons (IFNs) are a family of cytokines which are grouped into 2 classes viz. type I and type II. Type I interferon has antiviral activities while type II interferon has anti-bacterial, anti-parasitic and anti-fungal activities^23^. In addition, they have immunomodulatory responses making the IFNs very much essential for protecting the body from microbial infection. Accordingly, we have analysed expression of IFN-alpha and IFN-beta (Type-I) and IFN-gamma (Type-II) genes in blood cells of both transgenic and control birds so that we could assess the functionality of these genes in transgenic and control birds. Significant differences of expression of IFNB and IFNG between transgenic and control groups indicated that the body immune system was partially affected on account of transgene integration in the transgenic birds.

Besides blood cells, many serum biochemical parameters have been analysed in transgenic and control birds, which reflects general health status of the birds. The significantly lower serum cholesterol and triglycerides were observed in transgenic birds than that of the control ones both during 1st and 2nd generation. In case of ACACA transgenic birds, 21.2 and 18.5% lower level of the total cholesterol was detected in 1st and 2nd generation, respectively. The serum triglyceride was also 10.5 and 21.1% lower in ACACA transgenic bird in 1st and 2nd generation, respectively. In SREBP1 transgenic birds, 23.8 and 35.6% lower level of serum total cholesterol and triglycerides were observed during 1st generation. The lower level of cholesterol and triglyceride in the transgenic birds may be due to knocking down of ACACA and SREBP1 expression in transgenic birds. As SREBP1 acts as the transcription factor of the genes involved in de novo lipid biosynthetic path way, knocking down of SREBP1 showed better effect on lowering serum cholesterol and triglyceride contents in transgenic birds than that of ACACA transgenic birds.

Serum uric acid content differed between transgenic and control groups, where during 1st generation it was lower in transgenic group, but during 2nd generation, it was higher in transgenic birds. In addition, serum creatinine content was higher in transgenic birds than the control birds during 2nd generation. We suggest that the difference of serum uric acid and creatinine are specific to the bird’s own physiology and may not be directly associated with the knockdown of ACACA/SREBP1 expression. On account of higher growth of transgenic birds during 2nd generation, serum uric acid and creatinine level was higher than the control ones. Uric acid is normally synthesized by breaking down of purine nucleotides. In knock-down birds of 1st generation, low uric acid synthesis may be due to lower lipid synthesis associated to poor break down of purines. The results are very encouraging as lower uric acid synthesis is associated with good health condition of the transgenic birds.

As the cholesterol synthesis becomes affected due to knock down of ACACA and SREBP1 genes, it is critical to analyse the steroid hormones in transgenic and control birds. Accordingly, two important steroid hormones viz. progesterone and estrogen were estimated in serum of transgenic and control birds. The progesterone concentration was higher in transgenic birds compared to the control bird. During egg laying cycle, just before onset of ovulation, progesterone concentration becomes normally low and after ovulation it increases for a while^24^. However, estrogen concentration in transgenic birds was more or less similar to that in control birds. During follicular growth, estrogen level becomes high and it reaches to peak when oocytes become matured leading to LH surge for initiating ovulation^25^. Thus, estrogen becomes more critical with respect to ovulation and egg production^26^. But, the estrogen level in control and transgenic birds did not vary significantly during both 1st and 2nd generation. Though serum cholesterol content in transgenic birds were quite lower than the control birds, the secretion of these two steroid hormones were higher in transgenic birds than the control ones. Hence, we may suggest that the silencing of ACACA and SREBP1 expression did not have significant impact on synthesis of progesterone and estrogen in birds.

Egg production was numerically lower in SREBP1 transgenic birds than the control ones while egg production in ACACA transgenic and control birds did not differ significantly. Further, age at sexual maturity was also delayed in transgenic birds than the control ones. Endocrinological analysis revealed that in both SREBP1 and ACACA transgenic birds, progesterone concentration was significantly higher than that of control birds. On the other hand, estrogen concentration did not vary between transgenic and control birds. The progesterone and estrogen levels of birds during 1st generation was measured at 51 weeks of age while during 2nd generation, these hormones were estimated at 25 weeks of age indicating higher concentration of these hormones during later stage of egg laying cycle than early egg laying cycle.

In case of egg quality traits, yolk percentage was significantly lower in SREBP1 transgenic birds than that of control birds. Egg yolk contains 50% water, 30.6% lipids, 17% proteins, 0.6% carbohydrates and 1.7% minerals^27^. Overall the serum cholesterol and triglycerides contents in transgenic birds were lower than those of control birds. Thus, it may be possible that due to lower triglycerides and cholesterol content in serum, the yolk% become lower in eggs of transgenic birds, which were also reflected in egg cholesterol and triglyceride contents. In case of shell percentage, transgenic birds had higher shell content than that of control birds. Further, egg calcium level was also significantly higher in transgenic birds than the control ones. Literature reported that increase of serum calcium level correlates with worsening of lipid profile in human^28^. Thus, we may suggest that calcium metabolism is better when cholesterol content is low. Thus, calcium level in egg becomes numerically high leading to higher shell content in eggs of transgenic birds. Other minerals such as Zn, Mn and B were also higher in transgenic birds than the control birds. Thus, higher mineral contents in egg will be of very much useful for egg eating human being to supplement body with organic minerals for better biological activity. These important minerals may be of much use in persons suffering from mineral deficiencies. In addition, semen quality in transgenic birds differed from control ones. Though semen volume ejaculated from the transgenic cock was higher than control male, the individual mortality was lower in transgenic birds leading to lower MTT dye reduction in sperm of transgenic bird, which is an indicator of sperm fertility^29^. However, acrosomal integrity which is another indicator of sperm quality was similar both in transgenic and control male birds. It is inferred that ACACA transgenic shows by and large similar quality of sperm as has been in control one.

In this study, we have perfected the protocol to develop transgenic chicken through RNAi for knocking down the expression of ACACA and SREBP1 proteins, which minimized the total cholesterol and triglycerides in serum and eggs. We also assessed several physiological parameters such as growth, blood cells, blood parameters, serum biochemical profile, egg mineral contents, egg production and egg quality traits in transgenic and control birds.

## Methods

### Animals

The study was carried out in control broiler chicken line maintained at the experimental farm of ICAR-Directorate of Poultry Research, Hyderabad, India. A total of 106 hens were inseminated with the pooled processed semen collected from 28 cocks. A total of 150 fertile eggs were collected from the inseminated hens. The detail experimental design and hatching performance has been mentioned in Table 1. The whole study was approved by the Institute Animal Ethics Committee (IAEC) and Institute Biosafety Committee (IBSC) of ICAR-Directorate of Poultry Research, Hyderabad, India. All methods in the study were carried out in accordance with relevant guidelines and regulations of the Institute Animal Ethics Committee (IAEC) and Institute Biosafety Committee (IBSC). The work was also approved by the Review Committee on Genetic Manipulation (RCGM), Dept. of Biotechnology, Govt. of India. All the biosafety guidelines were followed while conducting the experiments. The study was carried out in compliance with the ARRIVE guidelines. The animal welfare measures like *ad lib* feeding, watering and management of the birds were taken care off during the experiment.

### Designing and cloning of shRNA molecules

**Figure 6:**
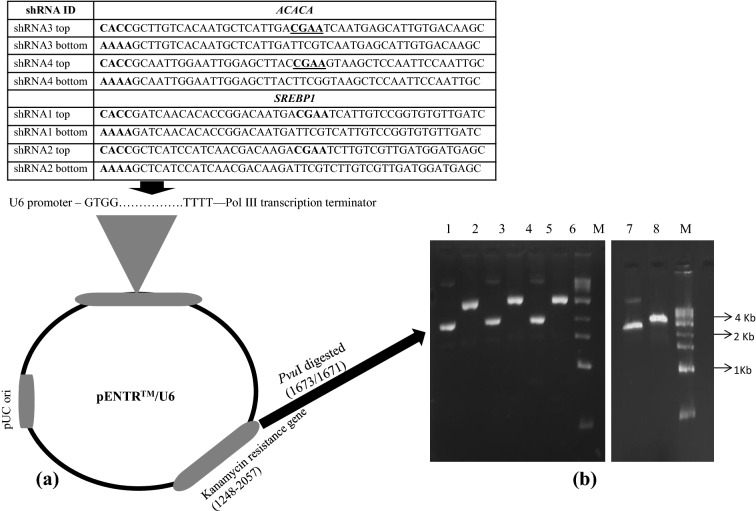
(**a**) pENTR/U6 entry vector where shRNA molecules were cloned. (**b**) shRNA cassette of ACACA and SREBP1 genes linearized by digestion with *Pvu*I enzyme. 1 = *Pvu*I digested shRNA3 cassette of ACACA; 3 = *Pvu*I digested shRNA4 cassette of ACACA; 5 = *Pvu*I digested shRNA1 cassette of SREBP1; 7 = *Pvu*I digested shRNA2 cassette of SREBP1; 2 = Undigested shRNA3 cassette of ACACA; 4 = Undigested shRNA4 cassette of ACACA; 6 = Undigested shRNA1 cassette of SREBP1; 8 = Undigested shRNA1 cassette of SREBP2; M = 1 Kb ladder DNA marker. The *Pvu*I restriction site (1673/1671) is present within the Kanamycin resistance gene (1248–2057) to neutralize the antibiotic resistance gene. The shRNA constructs have been mentioned in this table.

The shRNA molecules were designed from the coding sequence of chicken *ACACA* (Accession No. NC_006106) and *SREBP1* (Accession No. NC_006101) genes with Block-iT RNAi designer programme (https://rnaidesigner.thermofisher.com /rnaiexpress/) (Invitrogen). Two best shRNA molecules were identified based on our previous study conducted under cell culture system for *ACACA*^14^ and *SREBP1*^15^ genes. Double-stranded oligos encoding shRNA to the target genes were cloned in U6 promoter guided pENTR/U6 vector (Invitrogen) to prepare RNAi cassette. The entire RNAi cassettes were used as expression clones (Fig. 6a).

### Sperm mediated gene transfer (SMGT)

A total of 28 cocks of control broiler line were randomly selected for the experiment. The semen collected from all the cocks were pooled and centrifuged at 2000 g for 10 min to remove seminal plasma. The sperms were washed with PBS buffer (137 mM NaCl, 2.7 mM KCl, 10 mM Na_2_HPO_4_ and 2 mM KH_2_PO_4_) for 3–4 times. The numbers of sperms were counted in the Neubeur chamber. The live and dead sperms were evaluated after staining with Eosin-Nigrosin stain. The total sperms were divided into 5 groups consisting of 4 shRNAs groups and 1 control group (Only electrical impulse without DNA was provided to the sperm before insemination). All 4 RNAi cassettes were digested with *Pvu*I restriction enzyme (RE) (NEB Inc., Massachusetts, USA) at 37 °C for overnight (Fig. 6b). Then, the RE enzyme was inactivated by incubating digested DNA samples at 65 °C for 30 min at dry bath. The linearized recombinant DNA was quantified in Nanodrop spectrophotometer. After that, the sperms were transfected with 10 µg of each RNAi cassette of ACACA and SREBP1 genes by electroporation with Gene Pulser (Biorad) at 160 mV for 25 ms for 1 pulse. The transfected sperms containing 100 Million sperm in 0.25 ml PBS were inseminated to each hen under each treatment group. The same protocol of insemination was repeated on the consecutive days and eggs were collected for 2 days. The eggs were incubated at the incubator for 18 days at 98–100°F with 78–80% relative humidity and turning 6 times a day. Then, eggs were candled on 19th day and the fertile eggs were kept in the hatcher for 3 days at 98–100°F with 78–80% relative humidity. The chicks were hatched and all chicks were wing-banded with metal bands. The chicks were maintained in the brooder house on deep litter system upto 6 weeks of age by providing *ad lib* feeding, watering, lighting to warm the house and water sprinkling on the roof of the poultry shed during summer season. On 7th week, birds were kept in battery brooder and *ad lib* feeding and watering were provided. The birds were fed with 21% CP and 2800 ME energy upto 6 weeks of age and afterwards, 18% CP and 2900 ME was provided to the adult birds.

### Screening of positive transgenic birds by PCR, sequencing and Southern blotting

Blood samples were collected from all the birds of different treatment groups alongwith the control group. Genomic DNA was isolated from all the samples following standard protocol^30^. The primers designed on entry vector (Forward: 5′GGACTATCATATGCTTACCG3′ and Reverse: 5′-CAGGAAACAGCTATGAC-3′) to amplify the 293 bp entry vector fragment for screening of positive transgenic birds. The amplified products were run on 1% agarose gel. The presence of 293 bp band on the gel indicated the positive birds possessing the entry vector (Fig. 1a, b). The amplified products of the positive birds carrying entry vector were sequenced by Sanger’s di-deoxy chain termination method in ABI PRIZM 377 DNA sequencer (Perkin-Elmer, Waltham, MA) to confirm the presence of entry vector in the positive birds.

All the positive birds were also subjected to Southern blotting by digesting with *Apa*I restriction enzyme for confirmation. Probes were prepared from 293 bp fragment of pENTR/U6 vector backbone, which were labelled with biotin-streptavidin conjugated to alkaline phosphatase and detected with NBT/BCIP for spot hybridization (Biotin chromogenic detection kit, Thermo scientific, Cat. No. K0661).

### ELISA to detect ACACA and SREBP1 proteins in serum

The levels of ACACA and SREBP1 proteins in serum samples of knock down and control birds were measured by sandwich ELISA (enzyme-linked immuno-sorbent assay). Antibodies specific to ACACA and SREBP1 were pre-coated onto a 96-well plate (12 × 8 Well Strips) and blocked. Standards or test samples were added to the wells and were incubated at room temperature for 30 min. The primary antibodies specific to ACACA or SREBP1 were added, incubated at room temperature for 30 min followed by washing. Then, HRP-Peroxidase conjugate was added, incubated at room temperature for 30 min and unbound conjugate was removed by washing. The TMB substrate was added for enzymatic reaction at room temperature for 15 min at dark place where HRP generating a blue color product was changed to yellow after adding acidic stop solution (1 M HCl). The density of yellow coloration read by absorbance at 450 nm in ELISA reader was quantitatively proportional to the amount of sample ACACA or SREBP1 captured in the well. The absorbance values for ACACA or SREBP1 protein contents in the serum of transgenic and control group of birds estimated through ELISA were compared between transgenic and control groups, and between titres within each group following LSD statistical test (SPSS 20.0 software).

### Growth traits

Body weights of all the experimental birds at 1st generation including control were recorded at day1, 5th, 6th, 8th, 10th 20th, 32nd, 40th and 52nd week of age. During 2nd generation, body weights of birds were measured on day1, 2nd, 4th, 6th, 8th, 10th and 20th week of age.

### Blood cell profiles

Blood samples were collected from the transgenic and control birds at 26 weeks of age. All the blood cells were counted in hemocytometer for all the samples following protocol (Bhattacharya et al., 2019). Other haematological parameters viz. Haemoglobin% (Hb%), PCV, MCV, MCH and MCHC were measured in Automatic UBM FX 19 T hematology analyzer (Unitron Biomedicals, Bengaluru, India). The ESR of whole blood was measured following disposable ESR pipette-Westergren method (Recombigen Laboratories Pvt. Ltd., Delhi, India).

### Biochemical parameters

The blood samples without anti-coagulant were also collected in 1.5 ml Eppendorf tube and kept at room temperature at 45° slanting position for 6 h. Serum was collected from upper phase and kept in fresh Eppendorf tube. The serum samples were used to estimate triglyceride (identi triglyceride test kit) by Glycerol phosphate oxidase (GPO) method, total cholesterol (identi cholesterol test kit) by CHOD-POD method (Cholesterol oxidase peroxidise), LDL (identi identi Directr LDL cholesterol test kit) by Polymer detergent method and HDL content (identi Direct HDL cholesterol test kit) by Polymer detergent method in Turbochem 100 Manager Blood analyzer (CPC Diagnostics, Chennai, India) following Manufacturer’s instructions.

Blood urea content in serum was measured following GLDH-Urease method using ERBA urea (BUN) kit (Transasia Bio-Medicals Ltd., Solan, HP, India) in Robonik Prietest Touch Plus Biochemistry analyzer (Robonik Pvt. Ltd., Mumbai, India). Serum creatinine was measured following Jaffe’s method using ERBA Liquixx creatinine kit (Transasia Bio-Medicals Ltd., Solan, HP, India) in Robonik Prietest Touch Plus Biochemistry analyzer (Robonik Pvt. Ltd., Mumbai, India). Serum albumin was measured following BCG dye method using ERBA Liquixx albumin kit (Transasia Bio-Medicals Ltd., Solan, HP, India) in Robonik Prietest Touch Plus Biochemistry analyzer (Robonik Pvt. Ltd., Mumbai, India). Serum uric acid was measured following Uricase-Trinder End point method using ERBA Liquixx-M uric acid kit (Transasia Bio-Medicals Ltd., Solan, HP, India) in Robonik Prietest Touch Plus Biochemistry analyzer (Robonik Pvt. Ltd., Mumbai, India).

### Progesterone and estrogen estimation

The progesterone and estrogen in serum samples of both transgenic and control birds of first generation at the age of 51 weeks and the birds of second generation at the age of 25 weeks was estimated following Chemiluminescence Immunoassay (CLIA) using progesterone and estrogen kit (Siemens, Munich, Germany) in Advia Centaur XP apparatus (Seimens, Munich, Germany).

### Egg cholesterol and LDL estimation

The eggs were weighed, broken and both albumen and yolk were taken in stainless steel bowl. The bowl was kept in a hot air oven at 60 °C temperature for 4 days for drying. After drying, the whole content was triturated in a pastel and mortar. The total egg powder was also weighed. An amount of 0.1 g of egg powder was taken in 1.5 ml eppendorf tube. A volume of nine times of anhydrous ethanol was added to the tube and the mixture was mechanically homogenized for 30 s using vortex^31^. Then, the mixture was centrifuged at 2500*g* for 10 min at 4 °C. The supernatant was transferred to a fresh 1.5 ml centrifuge tube. A volume of 100 µl of supernatant was kept in the analysis vial and cholesterol content in egg was quantified in Turbochem 100 Manager Blood analyzer (CPC diagnostics, Chennai, India) using cholesterol estimation kit (identi cholesterol test kit; CPC diagnostics, Chennai, India). We also analyzed LDL content of egg in Turbochem 100 Manager Blood analyzer (CPC diagnostics, Chennai, India) with LDL estimation kit (identi Direct LDL cholesterol test kit; CPC diagnostics, Chennai, India) following Manufacturer’s instruction.

### Egg quality traits and minerals estimation

The internal egg quality traits such as albumin%, yolk%, shell%, Haugh unit and yolk colour index at 52 weeks of age were measured in egg analyzer (Orka Food Technology Ltd., Utah, USA). The whole eggs without egg shells were dried and powdered. A quantity of 1 g whole egg powder was taken in a volumetric flask. A volume of 20 ml HNO3 was added to the flask and a funnel was placed in the flask. The flasks were kept on a hot plate at 150–180 °C till the particles were dissolved in the acid. The sample was filtered using Whatman filter paper No. 42 and the volume of the content in the flask was made up to 50 ml with distilled water. Then, the samples were placed in iCAP7200 ICP OES Duo (Thermo Scientific, Massachusetts, USA) for estimation of egg minerals such as Cu, Fe, Zn, Mn, Mg, Cr, Ni, Ca, B and Se.

### Semen quality analysis

Semen was collected with a sterile glass funnel from a transgenic and a control cocks at 30 weeks of age following cloacal-abdominal massage method. The semen was evaluated for volume of semen, concentration of sperm, sperm mobility, live and dead sperm%, abnormal sperm%, acrosomal integrity and MTT dye reduction test for sperm fertilization ability. The volume of the semen ejaculate was measured by drawing the sample into a 1 ml syringe. The concentration of sperm was estimated by the method using a colorimeter (CL 157; Elico Ltd, Hyderabad, India) at wave length at 540 nm^32^. Sperm motility was subjectively assessed as percentage of progressively motile sperm by placing a drop of diluted semen on a clean, grease-free glass slide, overlaid with a coverslip, and examined at 20 × magnifications. Percentage of live and dead sperm was estimated by differential staining technique using eosin–nigrosin stain^33^. For counting abnormal sperm, glass slide smear was prepared from each sample and 200 sperm were counted in each slide for calculating abnormal sperm percentage. The intact acrosome (Acrosomal integerty) in sperm was assessed as described in the literature^34^. Briefly, 10 μl of diluted semen was mixed with 10 μl of stain solution [1% (W/V) rose Bengal stain, 1% (W/V) fast green FCF and 40% ethanol in citric acid (0.1 M) disodium phosphate (0.2 M) buffer (McIlvaine's, pH 7.2–7.3)] and kept for 70 s. A smear from the mixture was made on glass slide, dried and examined under microscope with high magnification (100X). The acrosomal caps were stained blue in acrosome-intact sperm, and absence of staining in the acrosome region of acrosome reacted sperm. A minimum of 200 sperm were counted in each smear sample for calculating the per cent acrosome-intact sperm. Tetrazolium dye 3-(4,5-dimethylthiazol-2-yl)-2,5-diphenyltetrazolium bromide (MTT) reduction test was carried out in duplicate tubes and absorbance was recorded using a colorimeter (CL 157; Elico Ltd, Hyderabad, India) at 570 nm^35^. The MTT-dye reduction test was performed to determine the fertilizing ability of the sperms.

### Realtime PCR

Blood sample of transgenic as well as control birds at the age of 26 weeks were collected in vacutainer tube. Total RNA was isolated from whole blood using Trizol following manufacturer’s instruction (Sigma). The total RNA was reverse transcribed with oligo dT primer to synthesize single stranded cDNA. To analyse the cellular stress in knock down and control birds, we employed two heat shock protein genes viz. hsp10 and hsp70.

The mRNA expression of target (Hsp70, Hsp10, IFN-alpha, IFN-beta and IFN-gamma) and reference gene (GAPDH) (Glyceraldehyde 3-phosphate dehydrogenase) in triplicate were quantified in thermal cycler Applied Biosystems® Step One Real Time PCR (Life Technologies) with Maxima SYBR Green/ROX qPCR Master Mix (Thermo Scientific). The primers designed and used for Hsp70, Hsp10, IFN-alpha, IFN-beta, IFN-gamma and GAPDH genes have been mentioned in Table 14. The threshold cycle (Ct) value of the target and the reference genes were determined from qPCR reactions. The mRNA expression of target gene was analyzed by comparative Ct method of relative quantification. The gene quantification was expressed as “n-fold up/down regulation of transcription” in relation to an internal control. The expression of the target gene was calibrated by that of the reference gene (GAPDH), at each time point and converted to the relative expression (fold of expression), as follows^36^:

$$ {\text{Fold\,of\,expression}} = {2}^{{ - \Delta \Delta {\text{Ct}}}} $$

where ∆Ct = Average Ct of target gene (Hsp70/Hsp10/IFN-alpha/IFN-beta/IFN-gamma)—Average Ct of reference gene (GAPDH)

$$ \Delta \Delta {\text{Ct}} = {\text{Average}}\,\Delta {\text{Ct\,of\,target\,sample}} - {\text{Average}}\,\Delta {\text{Ct\,of\,calibrator\,sample}} $$

**Table 14:** Primer sequences used for mRNA expression analysis of hsp70, hsp10, IFN-alpha, IFN-beta, IFN-gamma and GAPDH genes in whole blood of transgenic and control birds at the age of 26 weeks.

Gene	Primer sequence (5′–3′)	Amplicon size (bp)
IFN-Alpha	F: CTGCTCACGCTCCTTCTG R: GTGTCGTTGAAGGAGCAAG	170
IFN-Beta	F: CTCCTTCAGAATACGGCTC R: GTGTGTGGGCTGCTAAGC	164
IFN-Gamma	F: GGCGTGAAGAAGGTGAAAGA R: AGCTTCTGTAAGATGCTGAAGAG	107
hsp70	F:CAATGACAAGGGTCGCCTTA R:CCCTATCTCTGTTGGCTTCATC	95
hsp10	F: CGTAACCAAAGGAGGCATCA R: CACTGGATGAATCTCACCATCC	115
GAPDH	F: CTGCCGTCCTCTCTGGC R: GACAGTGCCCTTGAAGTG	119

The annealing temperature for realtime PCR reactions was 58 °C. The expressions of hsp70 and hsp10 indicates the stress shown by blood cells. The expressions of 3 immune response genes namely, IFN-alpha, IFN-beta and IFN-gamma show the immune status of transgenic and control birds.

### Statistical analysis

The effect of knock-down of *ACACA* and *SREBP1* genes on growth traits, blood profiles, serum biochemical parameters, egg production and quality traits, serum and egg cholesterol, serum progesterone and estrogen concentration were analysed by GLM procedure with SPSS20.0 software. The shRNA groups and sex of the birds were used as fixed effects for all the traits. The model used for analyzing effects was Y = µ + R_i_ + E_ijk_.

Where, Y = Trait; µ = Overall mean; Ri = Fixed effect of ith shRNA molecule; E_ijk_ = kth residual effect.

The Duncan’s multiple range test (DMRT) was performed to determine the effect of each group.

## Supplementary Information

Supplementary Information 1.

Supplementary Information 2.

## Data Availability

All the data have been included in this research article.
