# Supplementary material for: Silencing acetyl-CoA carboxylase A and sterol regulatory element-binding protein 1 genes through RNAi reduce serum and egg cholesterol in chicken
Source: Sci Rep. 2022 Jan 24;12:1191. doi: 10.1038/s41598-022-05204-z (PMC8786841; doi:10.1038/s41598-022-05204-z)
Supplement: Supplementary file 1 — Supplementary Information 1. [file 41598_2022_5204_MOESM1_ESM.doc]

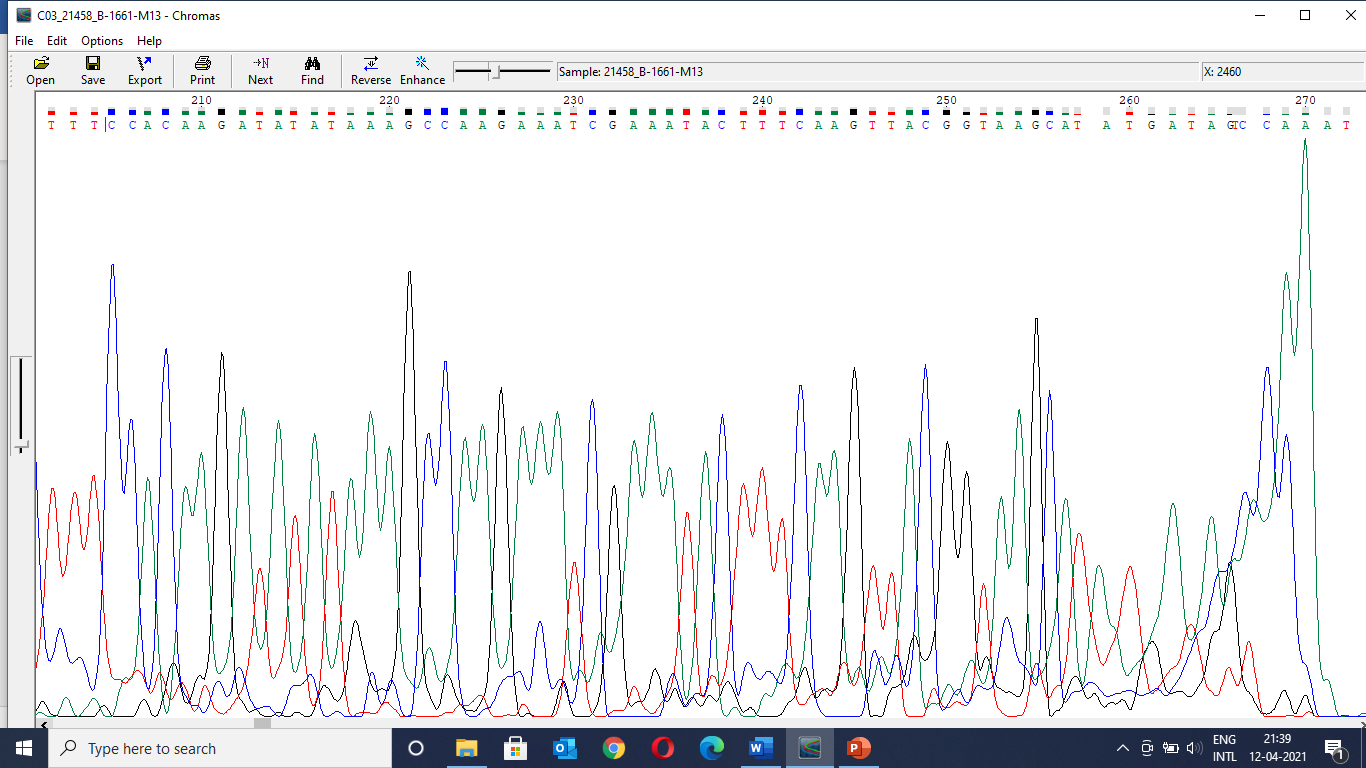


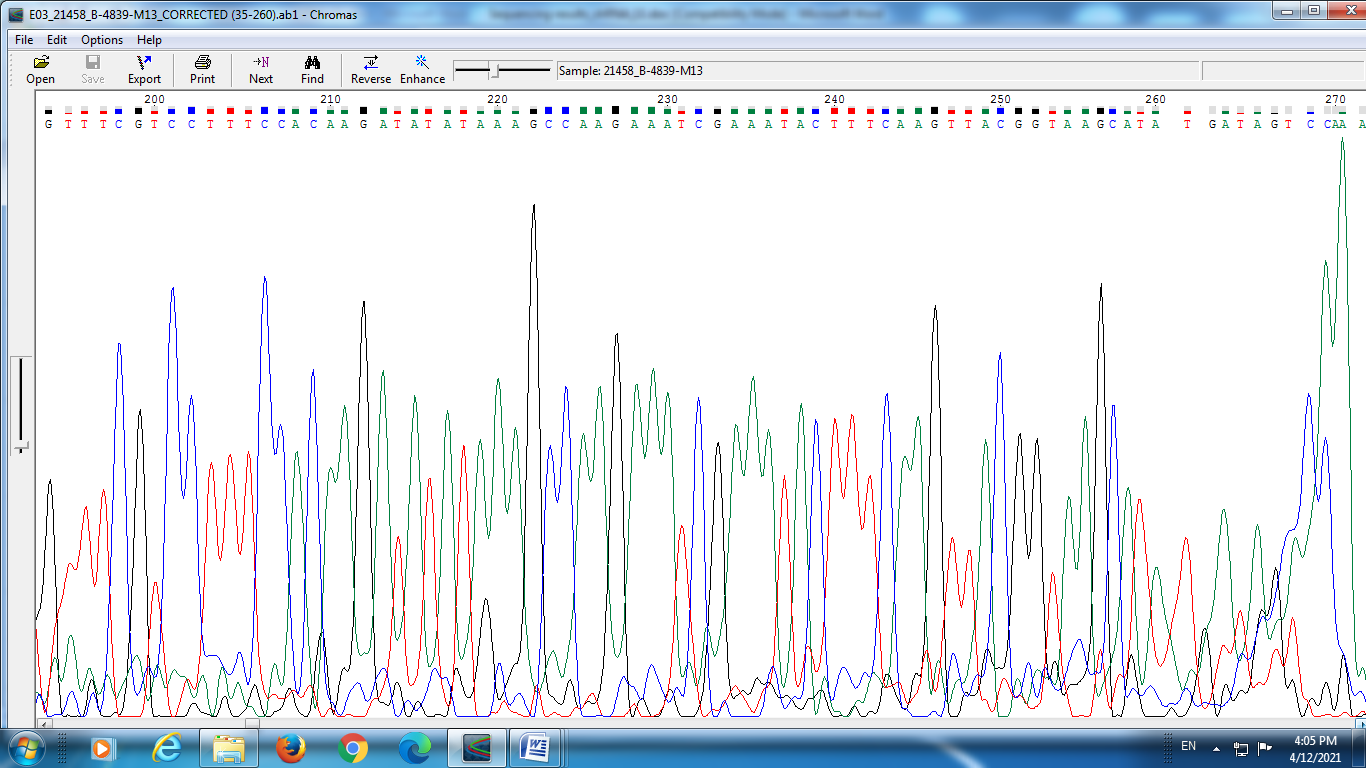


**Bird No. 4839**


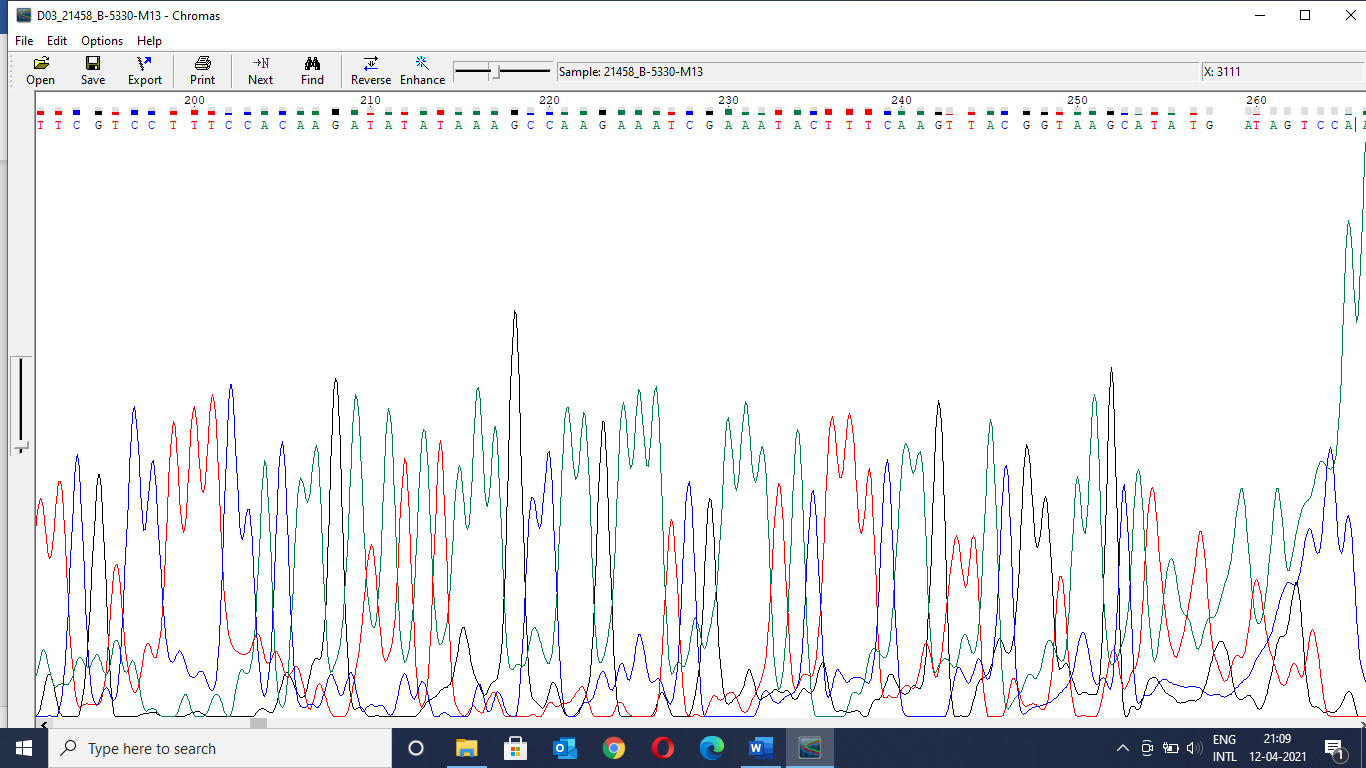


Vector sequence: TTTCGTCCTTTCCACAAGATATATAAAGCCAAGAAATCGAAATACTTTCAAGTTACGGTAAGCATATGATAGTCCA

**Bird No. 5330**

**Bird No. 1661**


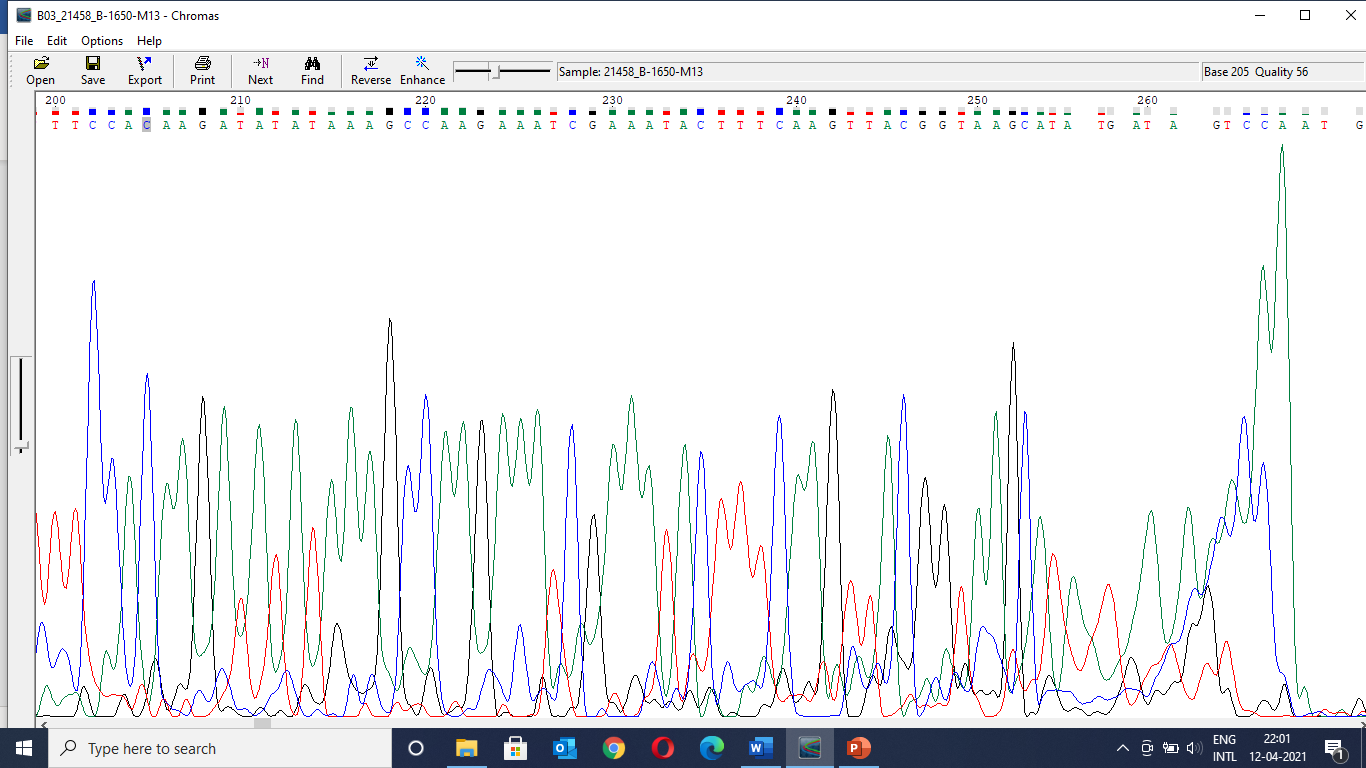


**Bird No. 1650**


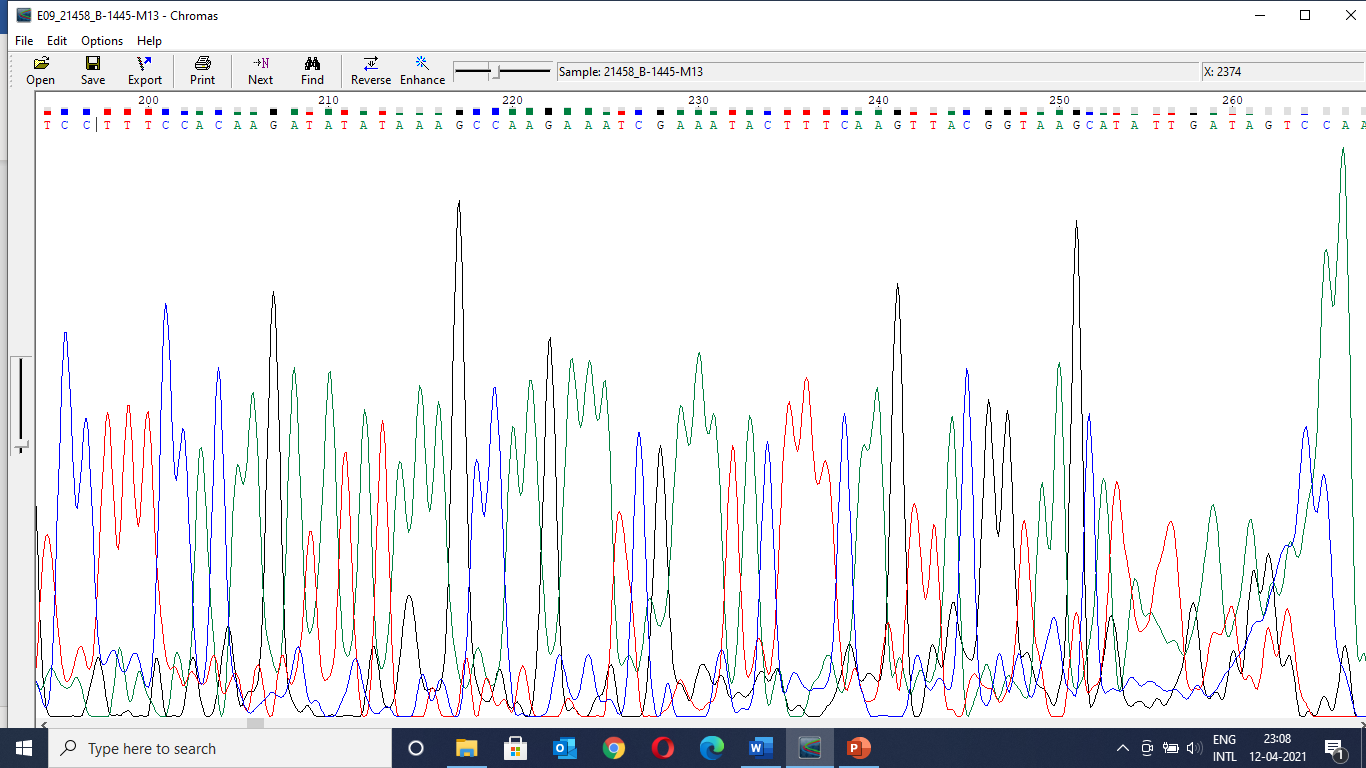


**Bird No. 1445**


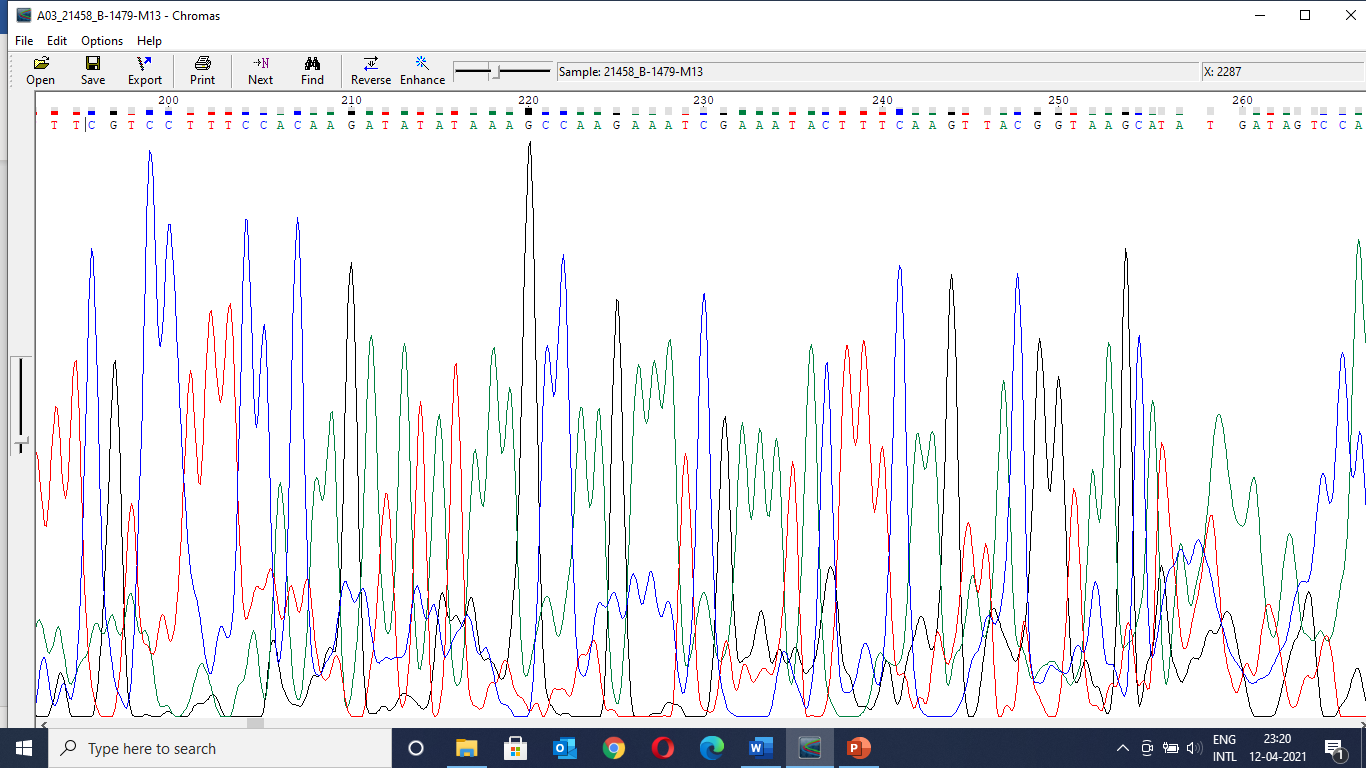


**Bird No. 1479**

Supplementary Figure 1. DNA sequence of entry vector fragment amplified from transgenic birds. Primer sequence has been under lined. Entry vector fragment sequence has been mentioned on top of chromatogram of Bird No. 4839. Presence of vector sequence in the bird indicates integration of entry vector in the genome of the transgenic birds.
