# Supplementary material for: Silencing acetyl-CoA carboxylase A and sterol regulatory element-binding protein 1 genes through RNAi reduce serum and egg cholesterol in chicken
Source: Sci Rep. 2022 Jan 24;12:1191. doi: 10.1038/s41598-022-05204-z (PMC8786841; doi:10.1038/s41598-022-05204-z)
Supplement: Supplementary file 2 — Supplementary Information 2. [file 41598_2022_5204_MOESM2_ESM.ppt]

## Slide 1
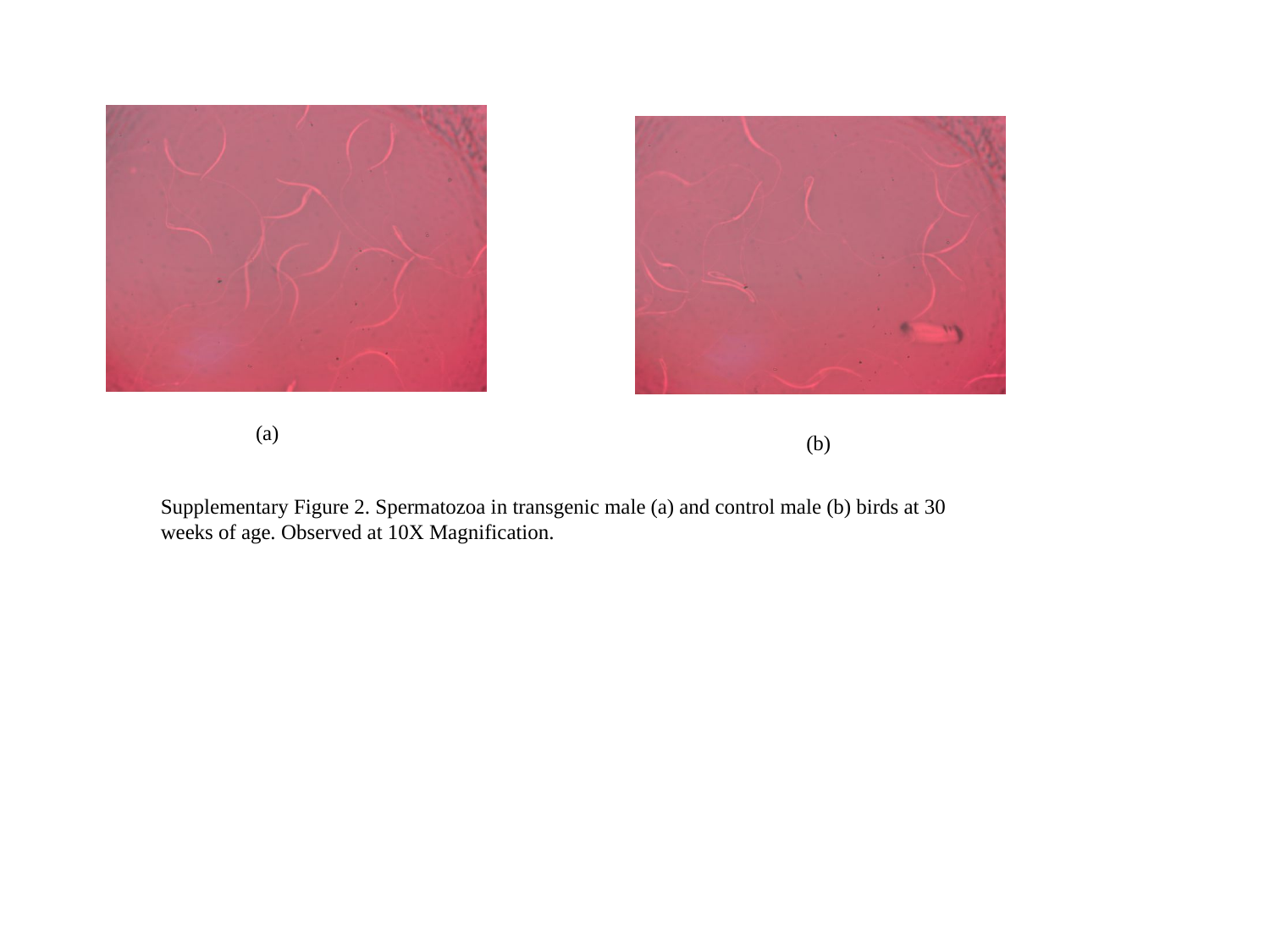

(a)
(b)
Supplementary Figure 2. Spermatozoa in transgenic male (a) and control male (b) birds at 30 weeks of age. Observed at 10X Magnification.

## Slide 2
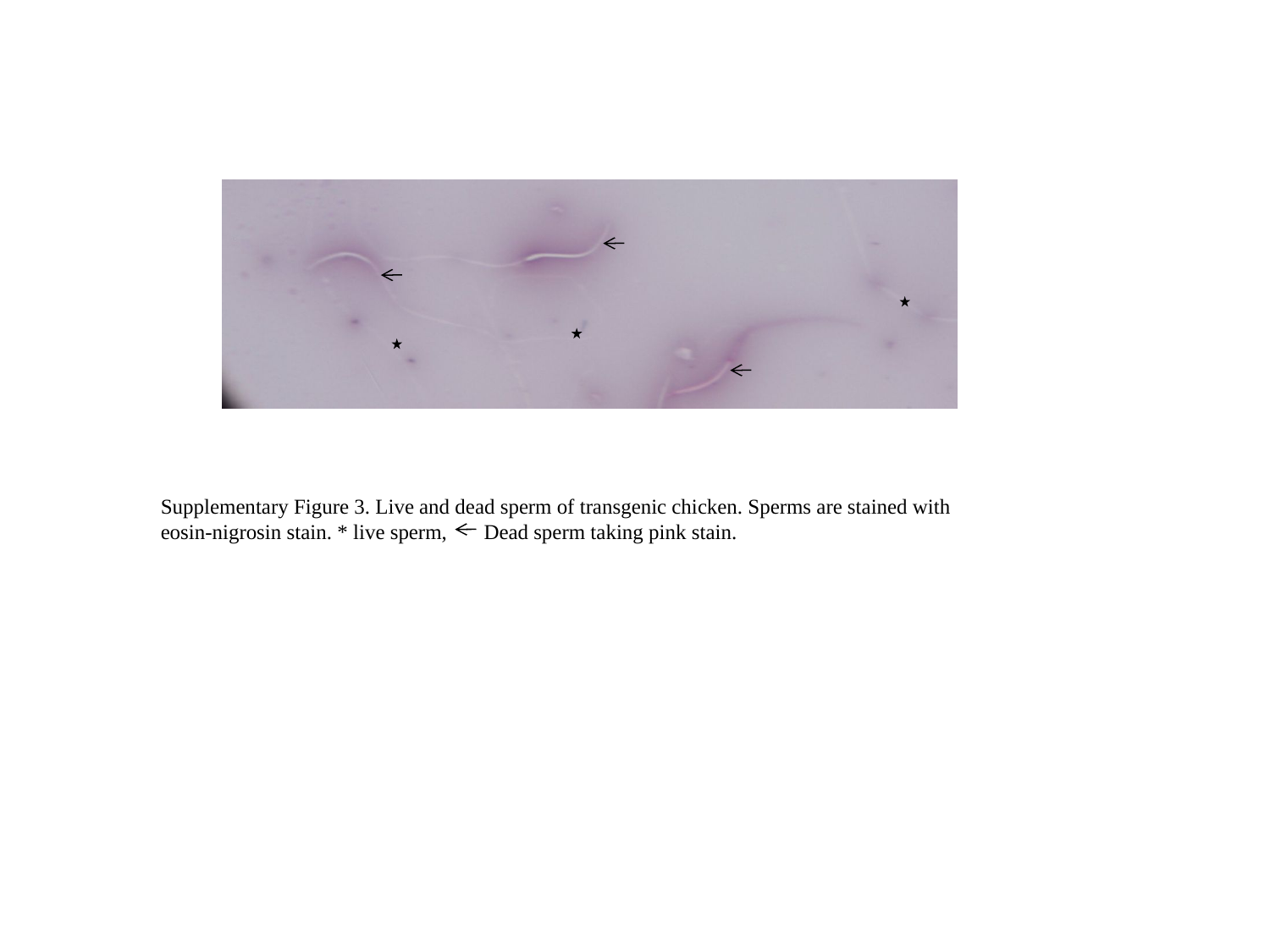

Supplementary Figure 3. Live and dead sperm of transgenic chicken. Sperms are stained with eosin-nigrosin stain. * live sperm, Dead sperm taking pink stain.
